# Structure, function, and inhibition of drug reactivating human gut microbial β-glucuronidases

**DOI:** 10.1038/s41598-018-36069-w

**Published:** 2019-01-29

**Authors:** Kristen A. Biernat, Samuel J. Pellock, Aadra P. Bhatt, Marissa M. Bivins, William G. Walton, Bich Ngoc T. Tran, Lianjie Wei, Michael C. Snider, Andrew P. Cesmat, Ashutosh Tripathy, Dorothy A. Erie, Matthew R. Redinbo

**Affiliations:** 1 Department of Chemistry, University of North Carolina at Chapel Hill, Chapel Hill, NC 27599 USA; 2 Department of Medicine, University of North Carolina at Chapel Hill, Chapel Hill, NC 27599 USA; 3 Department of Pharmacology, University of North Carolina at Chapel Hill, Chapel Hill, NC 27599 USA; 4 Department of Biochemistry and Biophysics, University of North Carolina at Chapel Hill, Chapel Hill, NC 27599 USA; 5 Department of Microbiology and Immunology, and Integrative Program for Biological and Genome Sciences, University of North Carolina at Chapel Hill, Chapel Hill, NC 27599 USA

## Abstract

Bacterial β-glucuronidase (GUS) enzymes cause drug toxicity by reversing Phase II glucuronidation in the gastrointestinal tract. While many human gut microbial GUS enzymes have been examined with model glucuronide substrates like *p*-nitrophenol-β-D-glucuronide (*p*NPG), the GUS orthologs that are most efficient at processing drug-glucuronides remain unclear. Here we present the crystal structures of GUS enzymes from human gut commensals *Lactobacillus rhamnosus*, *Ruminococcus gnavus*, and *Faecalibacterium prausnitzii* that possess an active site loop (Loop 1; L1) analogous to that found in *E. coli* GUS, which processes drug substrates. We also resolve the structure of the No Loop GUS from *Bacteroides dorei*. We then compare the *p*NPG and diclofenac glucuronide processing abilities of a panel of twelve structurally diverse GUS proteins, and find that the new L1 GUS enzymes presented here process small glucuronide substrates inefficiently compared to previously characterized L1 GUS enzymes like *E. coli* GUS. We further demonstrate that our GUS inhibitors, which are effective against some L1 enzymes, are not potent towards all. Our findings pinpoint active site structural features necessary for the processing of drug-glucuronide substrates and the inhibition of such processing.

## Introduction

The gastrointestinal (GI) microbiome harbours incredible metabolic potential and is intimately connected to human physiology. Possessing 150 times more genes than are found in the human genome, the gut microbiome encodes a vast number of enzymes that function in a variety of metabolic pathways, including the biosynthesis of essential vitamins and the breakdown of complex, non-digestible polysaccharides^1–4^. The gut microbiota has been termed both a “metabolic organ” and an “essential organ”, and it possesses a metabolic capacity that rivals that of the liver, which is critical to both anabolism and catabolism in the human host^5,6^.

Like the liver, the gut microbiota are capable of transforming xenobiotics such as pharmaceuticals, environmental pollutants, and dietary compounds ingested by humans^7^. However, the types of reactions performed by gut microbial enzymes are distinct from those performed by host liver enzymes. Drug metabolism enzymes in the liver transform relatively non-polar xenobiotics of low-molecular weight into molecules that are more polar and of a higher molecular weight, facilitating their excretion from the body^8^. Specifically, these reactions are carried out by Phase I enzymes, which introduce hydroxyl, thiol, and amine functional groups to the xenobiotic scaffold, and Phase II enzymes, which transfer glucuronide, sulphate, and glutathione moieties onto the Phase I functional groups or the xenobiotic scaffold^7,9^. In contrast, GI microbial enzymes perform hydrolytic and reductive transformations that are capable of reversing the Phase I and Phase II reactions performed by liver enzymes^10^. For this reason, the transformations carried out by microbial enzymes can drastically alter the pharmacological properties of xenobiotics.

Bacterial β-glucuronidase (GUS) proteins comprise one class of gut microbial enzymes that have been shown to reverse Phase II glucuronidation and, in doing so, cause the GI toxicity of several drugs^11^. This process has been extensively studied in connection with the colorectal and pancreatic cancer drug irinotecan and its active and toxic metabolite, SN-38^12,13^. Prior to excretion, SN-38 is sent to the liver where uridine diphosphate glucuronosyltransferase (UGT) enzymes attach a glucuronide group to the SN-38 scaffold, converting it to the inactive metabolite SN-38-glucuronide (SN-38-G), which is non-toxic. However, upon its delivery to the GI tract, gut microbial GUS enzymes hydrolyse SN-38-G and reactivate it back into its toxic form SN-38, which causes dose limiting diarrhoea^14,15^. In a similar fashion, NSAIDs have also been shown to cause small intestinal ulcers and inflammation, presumably due to the action of GUS enzymes that convert NSAID glucuronides back into their parent forms following Phase II glucuronidation^16^. In previous work, we have shown in mice that inhibitors selective for bacterial GUS alleviated SN-38 dose limiting diarrhoea and reduced the number of NSAID-induced small intestinal ulcers, further suggesting that GUS enzymes give rise to undesired GI side effects by reversing Phase II glucuronidation^17–19^.

**Figure 1:**
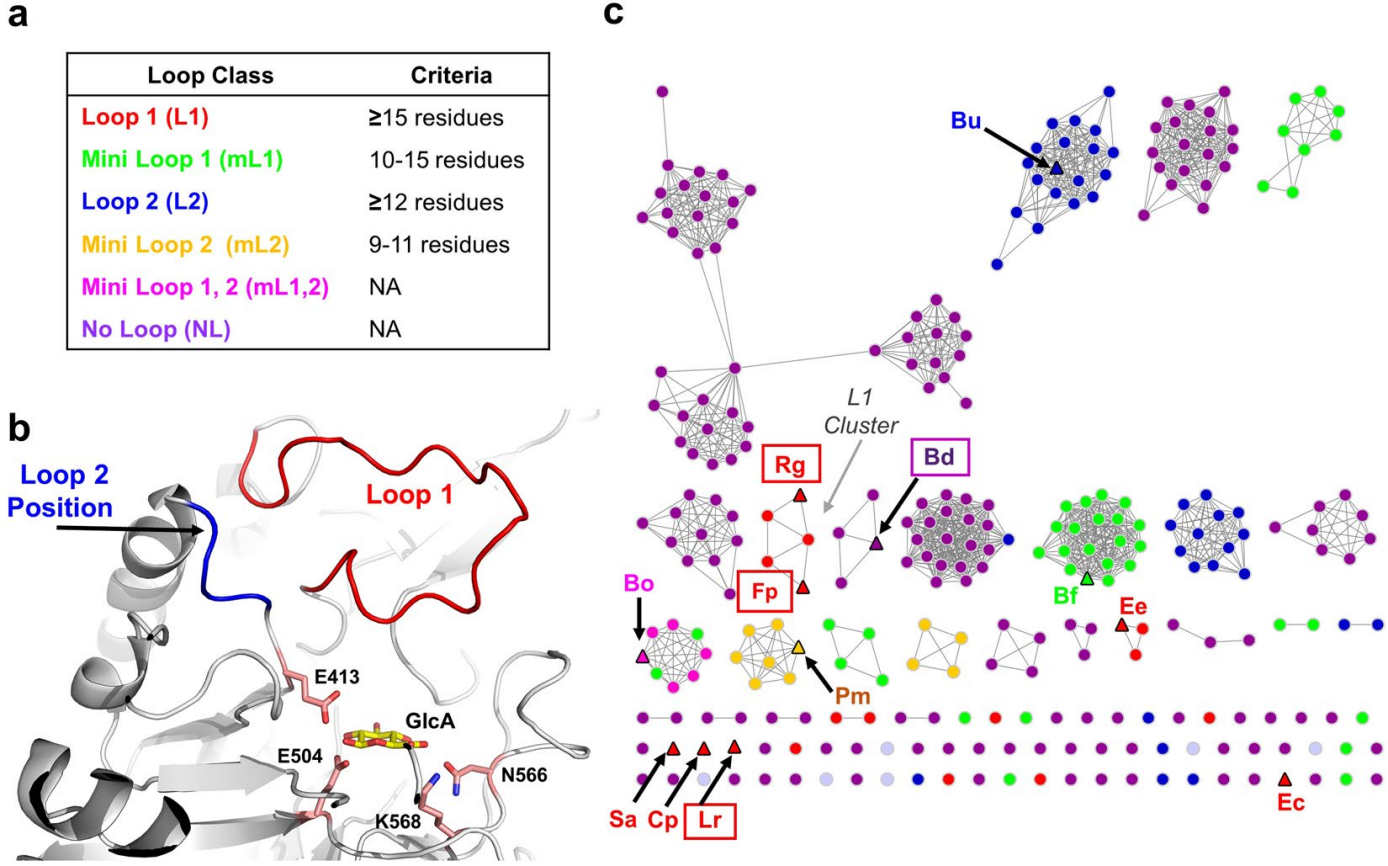
Loop classifications and clustering for the 279 GUS enzymes identified in the HMP database and the three novel L1 GUS enzymes. (**a**) Criteria for sorting GUS proteins into Loop classes as described in Pollet *et al*.^20^. (**b**) Loop 1 (*red*) and Loop 2 (*blue*) positions indicated in the *E. coli* GUS structure (PDB: 3LPG). Glucuronic acid (GlcA) is docked in the active site and shown in yellow. The catalytic E403 and E514 residues and the N566 and K568 residues that contact the carboxylic acid moiety of glucuronic acid are shown in light pink. (**c**) SSN for previously characterized GUS enzymes, the 279 GUS enzymes identified in the HMP database, and the novel L1 GUS sequences. GUS enzymes identified as Loop 1, Mini Loop 1, Loop 1, Mini Loop 2, Mini Loop 1,2, and No Loop are coloured as red, green, blue, yellow, pink, and purple, respectively. The GUS proteins previously characterized in Wallace *et al*. and Pollet *et al*. as well as the novel *Rg*GUS and *Lr*GUS proteins are indicated by triangles and labelled by the first letter of the genus followed by the first letter of the species of the bacterium name in which they are found; for example, Rg represents the GUS from *Ruminococcus gnavus*. GUS enzymes whose structures are reported in this paper are boxed. The cluster of Loop 1 enzymes is labelled as the “L1 Cluster”.

It is apparent that GUS enzymes are capable of hydrolysing a diverse array of glucuronides, but limited information is available on the specific types of GUS enzymes that are most efficient at processing drug glucuronides. In an attempt to gain insight into the structural and functional diversity of GUS enzymes, we recently reported an atlas of 279 unique GUS enzymes identified from the stool sample catalogue in the Human Microbiome Project (HMP) that clustered into six structural groups based on their active site loops, Loop 1 (L1), Mini Loop 1 (mL1), Loop 2 (L2), Mini Loop 2 (mL2), Mini Loop 1,2 (mL1,2), and No Loop (NL)^20^ (Fig. 1a–c). We further showed that representative GUS enzymes possessing a Loop 1 were capable of processing the small standard glucuronide substrate *p*-nitrophenol-β-D-glucuronide (*p*NPG) faster than non-L1 GUS enzymes^20^. We also found that two selective microbial GUS inhibitors, Inhibitor 1 and UNC10201652, were potent against the L1 *E. coli* GUS (*Ec*GUS) but did not inhibit the non-L1 GUS enzyme mL1 *Bacteroides fragilis* GUS (*Bf*GUS)^17,21,22^. From these data, we hypothesized that GUS enzymes possessing a Loop 1 efficiently process drug glucuronide substrates, such as SN-38-G and NSAID glucuronides, and are susceptible to inhibition by our GUS selective inhibitors.

To date, five L1 GUS enzymes, *Ec*GUS, *Eubacterium eligens* GUS (*Ee*GUS), *Streptococcus agalactiae* (*Sa*GUS), *Clostridium perfringens* GUS (*Cp*GUS), and *Faecalibacterium prausnitzii* (*Fp*GUS) have exhibited activity with *p*NPG, and all have been structurally characterized, with the exception of *Fp*GUS^17,20,21^. All four L1 GUS structures exhibited similar tertiary, quaternary, and active site architectures^17,21,22^. To determine whether any structural and functional differences exist among L1 GUS enzymes and to test our hypothesis that GUS enzymes containing a Loop 1 are the most efficient within the GUS family at processing drug glucuronides, we cloned, expressed, and purified two additional L1 GUS enzymes, *Lactobacillus rhamnosus* GUS (*Lr*GUS) and *Ruminococcus gnavus* GUS (*Rg*GUS). *Lactobacillus rhamnosus* was found to be adherent to healthy colon tissue in a patient biopsy obtained at UNC Hospitals (T. Keku, personal communication); thus, we chose to study a GUS from this bacterial species. *Ruminococcus gnavus* GUS was previously identified and examined for general biochemical properties^23^.

Here we present the crystal structures of the L1 GUS enzymes *Fp*GUS, *Lr*GUS, and *Rg*GUS, as well as the NL *Bacteroides dorei* (*Bd*GUS), and show that *Lr*GUS and *Rg*GUS exhibit unique active site features not previously seen in L1 GUS enzymes. We also determined the kinetic parameters k_cat_ and K_m_ for each L1 GUS enzyme and our panel of non-L1 GUS enzymes with the small standard substrate *p*NPG. Surprisingly, we found that *Fp*GUS, *Lr*GUS, and *Rg*GUS exhibited catalytic efficiencies 10 to 100-fold lower than the those of the L1 GUS enzymes previously characterized. We further demonstrate that while these three L1 GUS enzymes were not inhibited by our selective GUS inhibitors, NL *Bd*GUS was weakly inhibited, despite its lack of a Loop 1. We show that our panel of GUS enzymes differentially processed the NSAID metabolite diclofenac glucuronide (DCF-G) and that the relative cleavage rates were analogous to that observed for *p*NPG. Finally, we demonstrate that treating mice with diclofenac (DCF) increases GUS activity in faecal samples. These findings advance our understanding of the structure, function and inhibition of GUS enzymes. Furthermore, they suggest that the specific amino acid composition of Loop 1, as well as additional GUS structural features, likely play a role in the ability of GUS enzymes to cleave drug-glucuronides and in the efficacy of bacterial GUS inhibitors.

## Results

### Visualization of structure-function relationships across GUS proteins

Human gut microbial GUS enzymes have been previously shown to exhibit unique structures and functions^20^. To gain greater insight into the specific sequence-structure-function relationships among GUS proteins, particularly those belonging to the L1 class, we generated a sequence similarity network (SSN), which groups protein sequences into clusters and facilitates the analysis of functional relationships within protein families^24^. We utilized the 279 GUS protein sequences identified in the Human Microbiome Project (HMP) database to construct the SSN, as well as the sequences of the L1 GUS enzymes previously characterized (*Ec*GUS, *Ee*GUS, *Fp*GUS, *Cp*GUS, and *Sa*GUS), and the new L1 GUS sequences *Rg*GUS and *Lr*GUS^20,21^.

The GUS enzymes examined largely clustered based on their previously defined active site loop architectures: L1, mL1, L2, mL2, mL1,2, and NL (Fig. 1). Of the GUS enzymes that have already been examined both structurally and functionally, and are annotated on the SSN, we found that the non-L1 GUS enzymes, mL1 *Bf*GUS, L2 *Bacteroides uniformis* (*Bu*GUS), nL *Bd*GUS, mL1,2 *Bacteroides ovatus* (*Bo*GUS), and L2 *Parabacteroides merdae* (*Pm*GUS), clustered with GUS enzymes containing their same loop type (Fig. 1c). Three of the previously characterized L1 GUS enzymes, *Ec*GUS, *Sa*GUS, and *Cp*GUS, were singletons that did not group with any other GUS enzyme, while *Ee*GUS clustered with two other GUS enzymes from bacteria identified as *Eubacterium sp*. *Fp*GUS clustered with the new L1 *Rg*GUS and three other L1 GUS enzymes in a group we have termed as the “L1 Cluster” (Fig. 1c). One of the five GUS enzymes that compose the L1 Cluster was identified as a GUS from *Faecalibacterium prausnitzii* that shares 79% sequence identity to the previously characterized *Fp*GUS sequence^20^. The remaining GUS sequences that associated within the L1 Cluster were determined to belong to bacteria that could not currently be identified. Finally, the new L1 *Lr*GUS was a singleton and did not group with any other GUS enzyme.

To test our previous hypothesis that, among GUS family members, L1 GUS enzymes most efficiently process small glucuronide substrates, we cloned, expressed and purified two previously uncharacterized L1 GUS enzymes, *Rg*GUS and *Lr*GUS, for subsequent structural and functional studies. These two novel GUS enzymes, along with the ten GUS enzymes previously characterized, compose the panel of GUS enzymes examined below.

### Structural analysis

**Figure 2:**
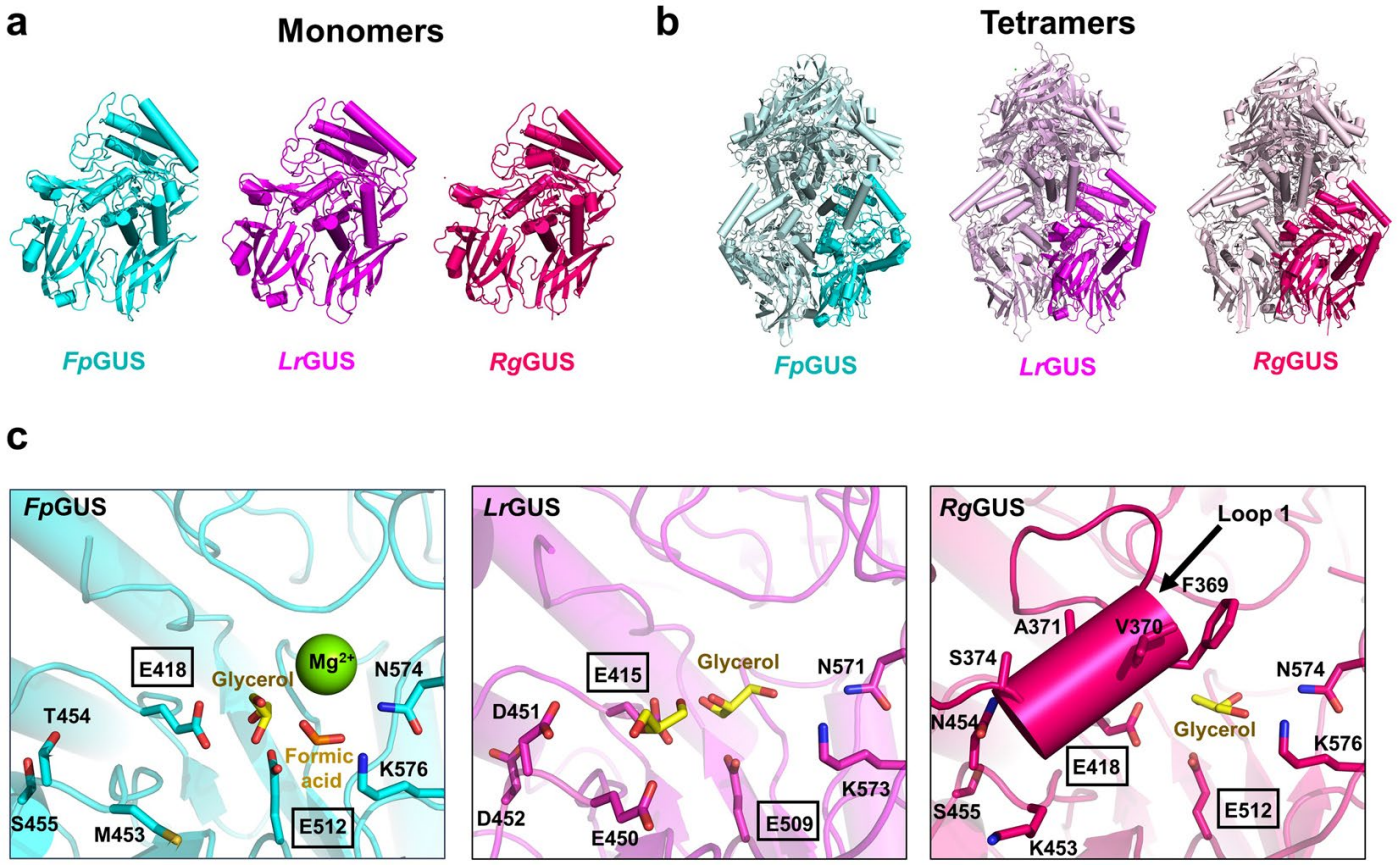
Crystal structures of L1 *Fp*GUS, *Lr*GUS, and *Rg*GUS enzymes. (**a**) Monomeric tertiary structures of *Fp*GUS (*cyan*), *Lr*GUS (*magenta*), and *Rg*GUS (*dark pink)*. (**b**) Tetrameric quaternary structures of *Fp*GUS, *Lr*GUS, and *Rg*GUS with a single monomer highlighted and identical monomers in its muted colour. (**c**) Active sites of *Fp*GUS, *Lr*GUS, and *Rg*GUS. An arrow points to the Loop 1 structure in *Rg*GUS. In *Fp*GUS and *Lr*GUS, the loop is disordered. A magnesium ion was built into the electron density within the *Fp*GUS active site. Given the crystallisation conditions (which contained magnesium formate), relatively solvent exposed active site observed in the structure, and the absence of unique coordinating residues, the ion is most likely an artefact from crystallization and is not expected to play a functional role in catalysis. Glycerol molecules are highlighted in yellow. Catalytic glutamates are boxed.

The structures of the L1 GUS enzymes *Ee*GUS (PDB: 6BJW), *Sa*GUS (PDB: 4JKL), *Cp*GUS (PDB: 4JKM), and *Ec*GUS (PDB: 3LPG) have been previously determined. To further examine the structural variability of L1 GUS enzymes, we determined the crystal structures of *Fp*GUS, *Lr*GUS, and *Rg*GUS (Fig. 2 and Table S1). The structures of these three new enzymes reveal highly similar tertiary structures (Fig. 2a) relative to one another, as well as to the previously determined L1 GUS enzymes (Fig. S1)^20,21^. *Fp*GUS, *Lr*GUS, and *Rg*GUS share a similar core fold with *Ec*GUS, with 1.7 Å root-mean-square deviation (rmsd), 1.7 Å rmsd, and 2.0 Å rmsd, respectively, over 576 Cα equivalent positions. The three new L1 GUS enzymes also retain the same tetramer organization compared to previously determined L1 structures, in which GUS monomers are in a “C-term-mediated” tetrameric state rather than a “square” tetrameric state, as exhibited by mL1 *Bf*GUS (PDB: 3CMG) and discussed below (Figs 2b and S2)^17,21^.

While *Lr*GUS, *Rg*GUS, and *Fp*GUS also exhibit similar tertiary and quaternary structures when compared to previously determined L1 GUS enzymes, *Lr*GUS and *Rg*GUS display unique features within their active sites. Specifically, *Lr*GUS possesses a patch of negatively charged residues, E450, D451, and D452, which we term the “EDD” motif (Figs 2c and S3a). These positions are generally occupied by polar or hydrophobic residues in our previously characterized L1 GUS enzymes, such as M453, T454, and S455 at the equivalent positions in *Fp*GUS, for example (Figs 2c and S3b). In the *Rg*GUS active site, six amino acids from the Loop 1 region of this enzyme were observed to adopt an alpha helix conformation that directly folds over the catalytic gorge (Fig. 2c). This structure represents the first instance in which Loop 1 adopts a secondary structural motif. Together, these data extend our knowledge regarding the active site structural variability sampled by human gut microbial GUS enzymes.

**Figure 3:**
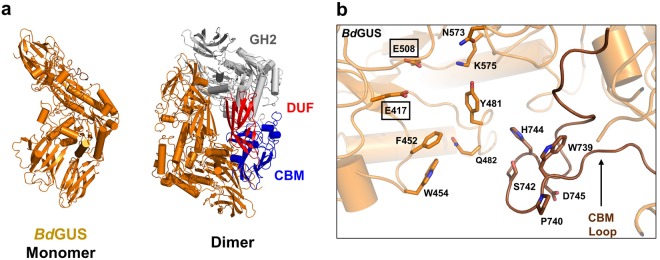
Crystal structure of NL *Bd*GUS. (**a**) Monomeric tertiary structure and dimeric quaternary structure of *Bd*GUS (*orange*). A single monomer is highlighted in orange and the identical monomer is shown with the glycosyl hydrolase 2 (GH2) in white, and the C-terminal domains (domain of unknown function, DUF; carbohydrate binding motif; CBM) in red and blue, respectively. (**b**) Active site of *Bd*GUS with the CBM loop indicated by an arrow. Catalytic glutamates are boxed.

To further advance our understanding of GUS structure and function we also determined the crystal structure of NL *Bd*GUS (Fig. 3 and Table S1). Like the L1 GUS structures, *Bd*GUS shares a similar core fold with *Ec*GUS, with 3.1 Å root-mean-square deviation (rmsd) over 528 Cα positions (Figs 3a and S1). Unlike the L1 GUS enzymes, *Bd*GUS is a dimer and possesses two additional domains at its C-terminus (Figs 3a and S2). Sequence analysis revealed that first C-terminal domain of *Bd*GUS is a “domain of unknown function” (DUF). The second C-terminal domain of *Bd*GUS is a member of the carbohydrate binding module (CBM) 57 family, based on malectin^25^. The presence of these C-terminal domains likely explains the unique quaternary arrangement of *Bd*GUS compared to that of other L1 GUS enzymes.

The tertiary and quaternary structure in *Bd*GUS is highly similar to that exhibited by *Bu*GUS, with a 2.3 Å root-mean-square deviation over 816 Cα positions (Fig. S4). Both contain one DUF and one CBM, and their dimers are arranged in identical configurations^20^. While *Bd*GUS does not possess a loop at the active site, as predicted by sequence analysis, it does contain a loop insert in the CBM that enters the active site and is not present in *Bu*GUS (Figs 3b and S4). Taken together, these structural data reveal new variability in active site features for the L1 GUS enzymes and a new understanding of the types of quaternary structures and active sites sampled by non-L1 GUS proteins.

### pNPG processing

L1 GUS enzymes have been previously shown to process the small standard substrate *p*NPG faster than non-L1 GUS enzymes^20^. To gain greater insight into the small molecule glucuronide processing capabilities of L1 GUS enzymes, we assessed the ability of *Lr*GUS and *Rg*GUS, which have not been previously characterized, to cleave *p*NPG by determining their k_cat_ and K_m_ at their optimal pH values. We determined the optimal pH values of *Lr*GUS and *Rg*GUS to be 4.5 and 6.5, respectively (Fig. S5). While *p*NPG hydrolysis by the remaining ten GUS enzymes in our panel have been previously assessed, these data represent either apparent k_cat_ values or k_cat_/K_m_ values determined at non-optimal pHs^17,20,21^. Therefore, we used the Michaelis-Menten equation to determine the k_cat_ and K_m_ values at the optimal pH of the remaining GUS enzymes analysed to generate a complete set of kinetic parameters suitable for comparison (Table 1 and Figs S5, S6, and S7). *Rg*GUS and *Bo*GUS exhibited complex substrate inhibition kinetics, and we were not able to fit these to established substrate inhibition models^26,27^ (Fig. S8). Consequently, apparent k_cat_ and K_m_ values were estimated at low *p*NPG concentrations at which inhibition was not observed (Figs S6 and S7). Future studies will explore the mechanistic and structural properties of this inhibition.

**Table 1:** Kinetic Parameters of *p*NPG Catalysis for GUS Enzymes.

Loop Class	GUS	Optimal pH	k_cat_ (s^−1^)	K_m_ (mM)	k_cat_/K_m_ (s^−1^mM^−1^)
L1	*E. coli*	7.4	120 ± 10^a^	0.13 ± 0.01^a^	920 ± 10^a^
L1	*E. eligens*	6.5	120 ± 8	0.223 ± 0.008	540 ± 20
L1	*S. agalactiae*	6.5	122 ± 3	0.17 ± 0.01	720 ± 30
L1	*C. perfringens*	6.5	57 ± 2	0.16 ± 0.02	360 ± 40
L1	*F. prausnitzii*	6.0	58 ± 4	2.2 ± 0.3	27 ± 2
L1	*L. rhamnosus*	4.5	10.0 ± 0.7	1.5 ± 0.1	6.7 ± 0.3
L1	*R. gnavus*	6.5	2.8 ± 0.3^b^	1.4 ± 0.2^b^	2.0 ± 0.1^b^
mL1	*B. fragilis*	5.0	22 ± 2	0.58 ± 0.09	39 ± 4
L2	*B. uniformis*	5.5	37 ± 2	0.54 ± 0.06	71 ± 6
mL2	*P. merdae*	5.5	0.088 ± 0.001	2.40 ± 0.07	0.0368 ± 0.0008
mL1,2	*B. ovatus*	5.0	0.48 ± 0.03^b^	1.38 ± 0.01^b^	0.34 ± 0.02^b^
NL	*B. dorei*	6.0	7.5 ± 0.4	1.4 ± 0.1	5.2 ± 0.5

Data are presented as the average of 3 biological replicates and errors are standard error of the mean (SEM). k_cat_ is catalytic rate; *K*_m_ is Michaelis constant; k_cat_/*K*_m_ is catalytic efficiency. Crystal structures reported in this study are underlined. ^a^Data were previously reported in Wallace *et al*.^17^. ^b^Data are reported as apparent k_cat_ and K_m_ values.

The L1 GUS enzymes *Ec*GUS, *Ee*GUS, and *Sa*GUS exhibited the highest turnover numbers (~120 s^−1^) and are the most efficient of the twelve GUS enzymes at processing *p*NPG, with catalytic efficiencies ranging from 540 to 920 s^−1^ mM^−1^. The L1 GUS enzymes *Cp*GUS and *Fp*GUS demonstrated moderate *p*NPG processing rates, exhibiting k_cat_ values of 57 s^−1^ and 58 s^−1^, respectively. However, *Fp*GUS exhibited the second highest K_m_ (2.2 mM) of all the GUS enzymes tested, resulting in a lower catalytic efficiency compared to that of *Ec*GUS, *Ee*GUS, *Sa*GUS, and *Cp*GUS. In addition, while *Fp*GUS was previously concluded to process *p*NPG faster than the non-L1 GUS enzymes *Bf*GUS and *Bu*GUS, determination of k_cat_ and K_m_ values reveal that *Fp*GUS demonstrates a lower catalytic efficiency than these two GUS enzymes^20^. Interestingly, the L1 GUS enzymes *Lr*GUS and *Rg*GUS exhibited much lower k_cat_ values (10.0 s^−1^ and 2.8 s^−1^, respectively) than the five other L1 GUS enzymes as well as K_m_ values ~10-fold higher than that of *Ec*GUS. *Lr*GUS and *Rg*GUS also demonstrated lower catalytic efficiencies than those of *Bf*GUS and *Bu*GUS, which are mL1 and L2 GUS enzymes, respectively. These results for *Lr*GUS and *Rg*GUS likely reflect their unique active site features, appearing to be electrostatically and sterically occluded from processing this small molecule glucuronide substrate.

*Pm*GUS and *Bo*GUS, which are mL2 and mL1,2 GUS enzymes, respectively, are even poorer than *Lr*GUS and *Rg*GUS in utilizing this substrate, exhibiting the lowest *p*NPG processing efficiencies observed, with k_cat_/K_m_ values of 3–4 orders of magnitude less than that of *Ec*GUS. The mL2 *Pm*GUS processed *p*NPG the slowest, with a k_cat_ value of 0.088 s^−1^ and exhibited the highest K_m_ value (2.40 mM). Thus, gut microbial GUS enzymes offer a range of active site architectures and activities toward a model small glucuronide substrate, likely reflecting the range of variable native substrates they likely evolved to process within the GI tract.

### GUS inhibition

Structural evidence suggests that the loop present in L1 GUS enzymes makes contacts with Inhibitor 1 and UNC10201652, stabilizing it within the active site^17,21,22^. We sought to assess the inhibition propensities of these inhibitors against both the L1 GUS and non-L1 GUS enzymes within our panel. To date, our inhibitors have only been tested with *p*NPG at pH 7.4^17,21^. However, the gut pH increases from approximately 5 in the proximal duodenum to about 7.4 in the colon^28^. Thus, we determined IC_50_ values for Inhibitor 1 and UNC10201652 against each GUS at pH 6.5 and 7.5 to sample two pH values in the GI tract (Figs S9 and S10).

We first tested whether we observed inhibition at 100 µM Inhibitor 1 or UNC10201652 at pH 6.5 and 7.5 for each GUS enzyme. However, due to their poor *p*NPG processing activity, exhibiting k_cat_ values of less than 1 s^−1^, *Pm*GUS and *Bo*GUS were not amenable for analysis. With the remaining ten enzymes, if 80% or greater inhibition was observed, an IC_50_ was determined. The L1 GUS enzymes *Ec*GUS and *Sa*GUS were inhibited by 80% or greater by 100 µM of Inhibitor 1 (Fig. S10a,b), while the remaining L1 GUS enzymes and the non-L1 GUS enzymes did not appear to be inhibited by 80% at 100 µM. Inhibitor 1 was most potent against *Ec*GUS and exhibited an IC_50_ value of ~2 µM at both pH 6.5 and 7.5 (Table 2 and Fig. S10a,b). For *Sa*GUS, Inhibitor 1 demonstrated an IC_50_ value of >25 µM (Fig. S10a,b) The potency of Inhibitor 1 did not appear to be affected by pH for either *Ec*GUS or *Sa*GUS (Table 2 and Fig. S10a,b).

**Table 2:** Potency of Inhibitors Toward a Range of Gut Microbial GUS Enzymes.

Loop Class	GUS	Inhibitor 1 (IC_50_, nM)		UNC10201652 (IC_50_, nM)	
		pH 6.5	pH 7.5	pH 6.5	pH 7.5
L1	*E. coli*	2000 ± 200	1900 ± 100	310 ± 20	100 ± 8
L1	*E. eligens*	ND	ND	1900 ± 100	410 ± 30
L1	*S. agalactiae*	>25000	>25000	450 ± 30	133 ± 3
L1	*C. perfringens*	ND	ND	160 ± 10	26 ± 3
L1	*F. prausnitzii*	ND	ND	ND	>3000
L1	*L. rhamnosus*	ND	ND	ND	ND
L1	*R. gnavus*	ND	ND	ND	ND
mL1	*B. fragilis*	ND	ND	ND	ND
L2	*B. uniformis*	ND	ND	ND	ND
mL2	*P. merdae*	NM	NM	ND	NM
mL1,2	*B. ovartus*	NM	NM	NM	NM
NL	*B. dorei*	ND	ND	ND	>25000

Uncertainties for IC_50_ values in nM are presented as the average of 3 biological replicates and errors are SEM. ND means not determined because no inhibition was observed up to 100 µM. NM means not measured because these enzymes showed k_cat_ values for *p*NPG cleavage of less than 1 sec^−1^.

At 100 µM UNC10201652, the L1 GUS enzymes *Ec*GUS, *Ee*GUS, *Sa*GUS, and *Cp*GUS were inhibited at both pH 6.5 and 7.5 (Fig. S10c,d). In contrast, *Fp*GUS and *Bd*GUS were inhibited at pH 7.5 but did not show an inhibition of 80% or greater at pH 6.5 (Fig. S10c,d). UNC10201652 was most potent against *Cp*GUS with an IC_50_ value of 26 nM at pH 7.5 and demonstrated nanomolar IC_50_ values for *Ec*GUS and *Sa*GUS at pH 7.5 (100 nM and 133 nM, respectively) (Table 2). Of all the L1 GUS enzymes tested, UNC10201652 was least potent towards *Fp*GUS, with a predicted IC_50_ value of >3 µM at pH 7.5 (Fig. S10c). Unlike Inhibitor 1, the potency of UNC10201652 decreased with decreasing pH for all enzymes tested (Table 2). The decrease in UNC10201652 potency appeared to vary with each enzyme tested. For *Ec*GUS, the potency decreased by 3-fold from pH 7.5 to pH 6.5, whereas the potency decreased by 6-fold for *Cp*GUS as the pH dropped from 7.5 to 6.5. Finally, despite its lack of an active site loop, the NL *Bd*GUS was found to be weakly inhibited at pH 7.5 by UNC10201652, with a predicted IC_50_ value of >25 µM (Fig. S10c). Thus, this compound appears to be capable of associating with gut microbial GUS enzymes via contacts not exclusively limited to Loop 1-mediated interactions.

### DCF-G processing

While *p*NPG serves as a useful tool to assess the glucuronide processing capabilities of GUS enzymes, it is not a substrate present in the GI tract. Given our hypothesis that L1 GUS enzymes efficiently process drug glucuronides, we determined using ultra performance liquid chromatography (UPLC) the cleavage rates of the novel L1 GUS enzymes *Fp*GUS, *Lr*GUS, *Rg*GUS, as well as the ten additional enzymes in our GUS panel, with the physiologically relevant glucuronide metabolite of the NSAID diclofenac, DCF-G (Table 3 and Fig. S11).

**Table 3:** Apparent k_cat_ for Various GUS Enzymes with DCF-G.

Loop Class	GUS	Optimal pH	Apparent k_cat_ (s^−1^)
L1	*E. coli*	7.4	30.7 ± 0.7
L1	*E. eligens*	6.5	138 ± 2
L1	*S. agalactiae*	6.5	97 ± 5
L1	*C. perfringens*	6.5	61.5 ± 0.6
L1	*F. prausnitzii*	6.0	32 ± 3
L1	*L. rhamnosus*	4.5	10 ± 1
L1	*R. gnavus*	6.5	24.0 ± 0.5
mL1	*B. fragilis*	5.0	15.1 ± 0.5
L2	*B. uniformis*	5.5	10 ± 1
mL2	*P. merdae*	5.5	0.034 ± 0.001
mL1,2	*B. ovatus*	5.0	1.64 ± 0.04
NL	*B. dorei*	6.0	9.8 ± 0.6

Data are presented as the average of 3 biological replicates and errors are SEM.

Analogous to their *p*NPG processing rates, *Lr*GUS and *Rg*GUS were the slowest L1 GUS enzymes to hydrolyse DCF-G and exhibited apparent k_cat_ values of 10 s^−1^ and 24.0 s^−1^, respectively, at a substrate concentration of 400 µM. In comparison, *Ee*GUS and *Sa*GUS hydrolysed DCF-G the fastest with an apparent k_cat_ of 138 s^−1^ and 97 s^−1^, respectively. *Ec*GUS, one of the three fastest GUS enzymes to process *p*NPG, cleaved DCF-G faster than *Lr*GUS and *Rg*GUS but slower than the other L1 GUS enzymes, exhibiting a k_cat_ of 30.7 s^−1^.

The non-L1 GUS enzymes processed DCF-G slower than all of the L1 GUS enzymes tested, with the exception of *Bf*GUS, which cleaved DCF-G at a rate of 15.1 s^−1^, and *Bu*GUS, which cleaved DCF-G at a rate equal to that of *Lr*GUS. *Pm*GUS processed DCF-G the slowest of all of the GUS enzymes tested, exhibiting the apparent k_cat_ value of 0.034 s^−1^. Taken together, these data demonstrate that overall trends with small molecule glucuronide substrates are maintained when comparing *p*NPG and DCF-G and suggest that L1 GUS enzymes catalyse the hydrolysis of small molecule glucuronides more efficiently than other classes of GUS enzymes. Specifically, these data highlight the fact that *Ee*GUS, *Cp*GUS and *Sa*GUS efficiently reactivate the inactive NSAID metabolite diclofenac-glucuronide back to the active and GI-toxic NSAID diclofenac, and are effectively inhibited by UNC10201652. Thus, not all L1 GUS enzymes process this small molecule drug glucuronide well, but those that do are inhibited *in vitro* by UNC10201652.

### Glucuronide processing by faecal extracts

The data presented above show that *p*NPG and DCF-G are processed by gut microbial GUS enzymes *in vitro*. To examine whether the GUS enzymes are also able to cleave a small glucuronide substrate *in vivo*, we incubated *p*NPG with the faecal extracts collected from four adult mice. *p*NPG processing was observed in all faecal samples, suggesting that the mouse intestine harbours GUS enzymes capable of processing drug glucuronide substrates (Fig. S12). To determine how DCF-G treatment alters the glucuronide processing activity of these GUS enzymes, we treated the mice with DCF-G and collected their faecal samples after 12 hours. Interestingly, DCF-G treatment increased *p*NPG processing activity in faecal extracts, which may be indicative of either an expansion of bacteria encoding DCF-G reactivating GUS enzymes, or an upregulated expression of these enzymes in specific gut microbial species following DCF-G treatment (Fig. S12). Taken together, these results support the conclusion that faecal samples contain GUS activity, and that treatment with a drug known to be glucuronidated, DCF in this case, increases GUS activity compared to pre-treatment levels.

## Discussion

### L1 GUS distribution across the SSN points to distinct substrate specificities

We previously demonstrated that a small set of L1 GUS enzymes are capable of processing small glucuronide substrates and are susceptible to selective GUS inhibition, while non-L1 GUS enzymes process larger glucuronide-containing substrates and are not effectively inhibited^20,21^. Here, we extend these investigations by examining the structure and function of two novel L1 GUS enzymes, as well as a panel of ten additional L1 and non-L1 GUS enzymes. By creating an SSN using the 279 GUS enzymes identified from the HMP, we found that while L1 GUS enzymes characterized in this paper retained highly similar tertiary and quaternary structures to previously characterized GUS enzymes, they do not cluster together (Fig. 1c). Further, *Ec*GUS, *Sa*GUS, *Cp*GUS, and *Lr*GUS did not cluster with any other L1 or non-L1 GUS enzyme. Given the fact that these L1 GUS enzymes displayed a range of DCF-G and *p*NPG cleavage rates, the separation of L1 GUS enzymes across the SSN may indicate that they possess distinct substrate specificities. These observations demonstrate the utility of SSNs to predict functional characteristics within large enzyme families encoded by the gut microbiome.

### Active site loop architecture is important in the efficient processing of small glucuronide substrates

**Figure 4:**
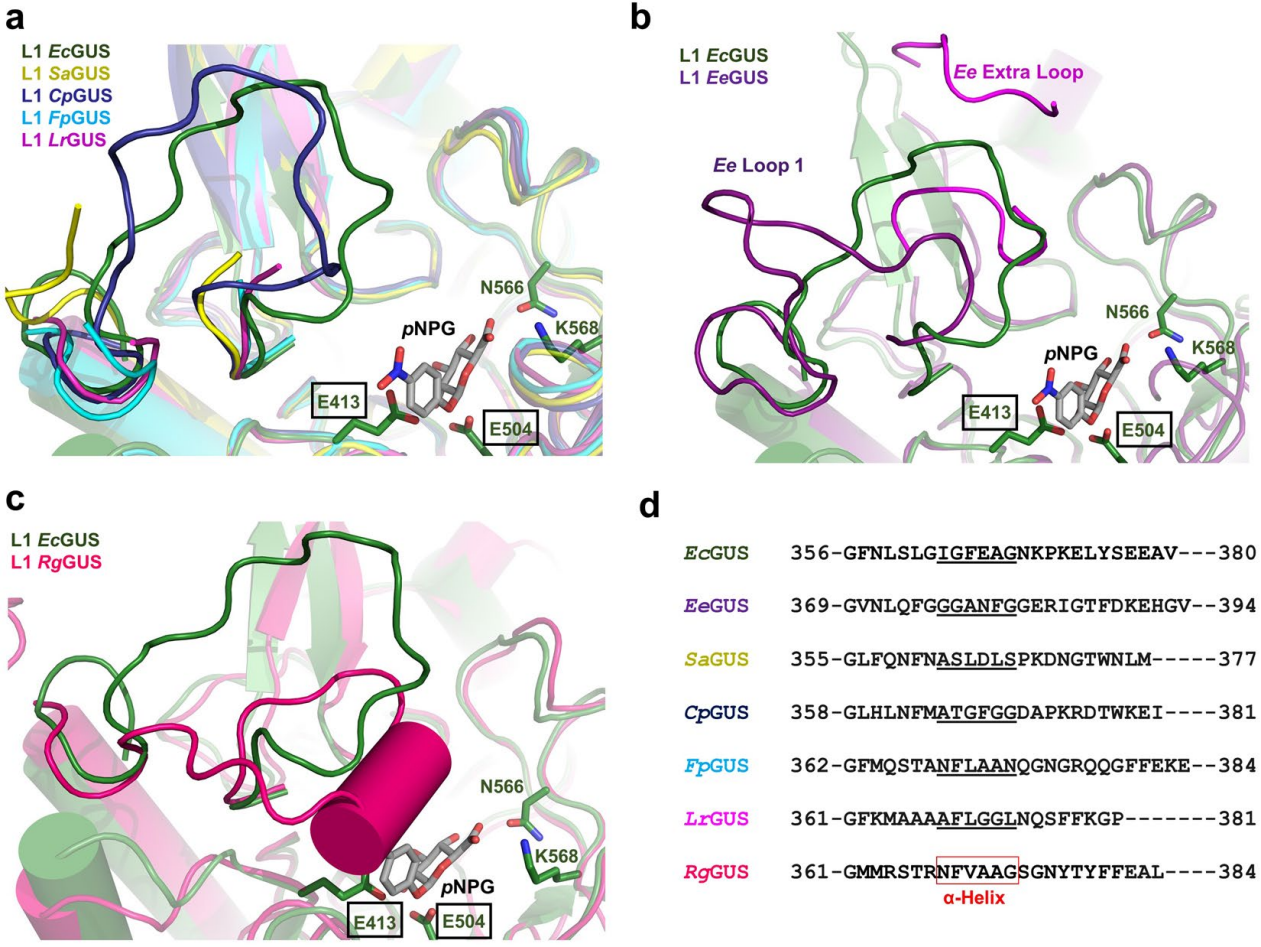
Alignment of the Loop 1 regions in L1 GUS enzymes. (**a**) Overlay of *Ec*GUS (3LPG; *dark green*) with *Sa*GUS (4JKK; *yellow*), *Cp*GUS (4JKM; *indigo*), *Fp*GUS (*cyan*), and *Lr*GUS (*magenta*), with *p*NPG shown docked into the active site of *Ec*GUS. (**b**) Overlay of *Ec*GUS and *Ee*GUS (purple). The canonical Loop 1 is shown in dark purple. The additional active site proximal loop, termed “Ee Extra Loop”, is shown in magenta. Residues G152 through G156 were not previously built and are not shown. (**c**) Overlay of *Ec*GUS and *Rg*GUS (dark pink) (**d**) Sequence alignment of the Loop 1 regions from the seven L1 GUS enzymes characterized in this study. Amino acids that form the alpha helix in the Loop 1 of *Rg*GUS are boxed in red. Amino acids that correspond to the alpha helix position in the Loop 1 of *Rg*GUS are underlined.

**Figure 5:**
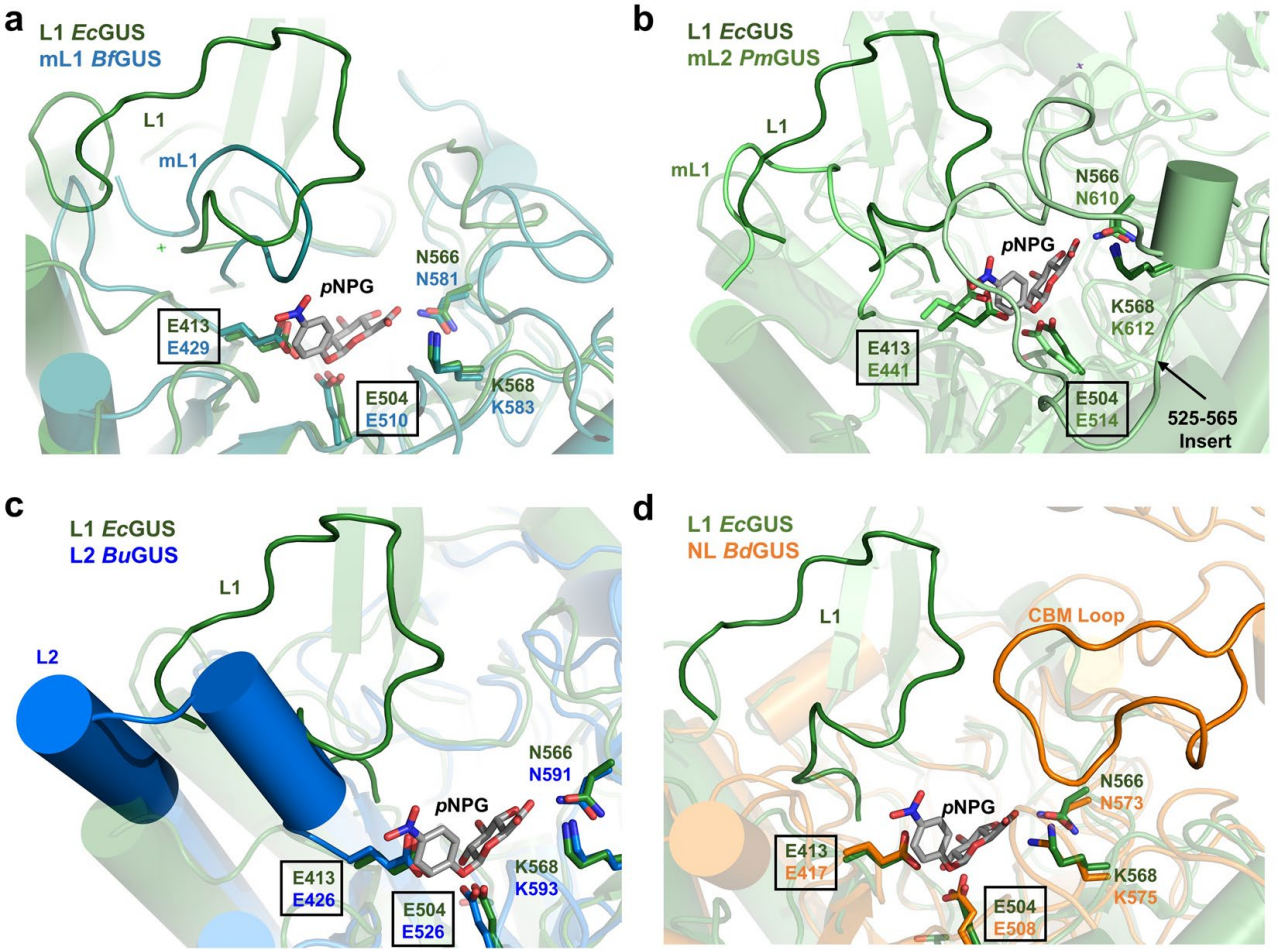
Structural alignments of the loop regions in non-L1 GUS enzymes. (**a**) Overlay of *Ec*GUS (*dark green*) with *Bf*GUS (*teal*). (**b**) Overlay of *Ec*GUS with *Pm*GUS (*light green*). (**c**) Overlay of *Ec*GUS with *Bu*GUS (*blue*). (**d**) Overlay of *Ec*GUS with *Bd*GUS (*orange*).

To explain the range of catalytic efficiencies exhibited by our panel of GUS enzymes, particularly the poor processing activity demonstrated by the new L1 GUS enzymes, we manually docked *p*NPG into the active site of each GUS enzyme (Figs 4 and 5). Given the diversity of the active site loops within the GUS family, Loop 1 position and length likely influence the ability of GUS enzymes to process *p*NPG and other small glucuronide substrates. Indeed, the L1 GUS enzymes *Ec*GUS, *Ee*GUS, *Sa*GUS, and *Cp*GUS demonstrated the highest k_cat_/K_m_ values compared to the non-L1 GUS enzymes (Table 2). The length (>15 amino acids) and position of Loop 1 in these enzymes appear to be suitable for stabilizing *p*NPG in their active sites and provide an explanation as to why these enzymes exhibited low K_m_ values (Table 1 and Fig. 4).

By contrast, the non-L1 GUS enzymes possess active site loops distinct in position or length compared to Loop 1. For example, mL1 *Bf*GUS possesses a Mini Loop 1 (Fig. 5a). *p*NPG docking reveals that the Mini Loop 1 of *Bf*GUS is too short to make productive contacts with *p*NPG (Fig. 5a). This observation suggests an explanation for why GUS enzymes possessing shorter loops between 10 and 15 residues, such as *Bf*GUS, *Bo*GUS, and *Pm*GUS, poorly process *p*NPG. As observed in the crystal structure of mL2 *Pm*GUS, a loop insert 41 residues in length occupies the active site and appears to clash with *p*NPG (Fig. 5b). As such, this loop insert may also explain why *Pm*GUS displays a high K_m_ value (2.40 ± 0.07 mM) for *p*NPG (Table 1).

While *Bu*GUS does not possess a shorter active site loop, the structure of this active site loop appears to explain why it does not process *p*NPG as efficiently as Loop 1 GUS enzymes. As reported in Pollet *et al*., the Loop 2 in *Bu*GUS adopts an extended alpha helix structure (Fig. 5c)^20^. Therefore, in this state it is not capable of folding over the active site to make contacts with *p*NPG. In addition, NL *Bd*GUS also lacks an active site loop; in contrast to *Bu*GUS, however, it does possess a loop in its CBM domain that enters the *Bd*GUS active site (Figs 3b and 5d). Despite this extra CBM loop, the absence of a loop ~15 residues in length that resides at the Loop 1 position may explain why *Bu*GUS, *Bd*GUS, and other non-L1 GUS enzymes exhibited higher K_m_ values for *p*NPG compared to *Ec*GUS, *Ee*GUS, *Sa*GUS, and *Cp*GUS.

Importantly, however, not all L1 GUS enzymes exhibited efficient k_cat_/K_m_ values. The three new L1 GUS enzymes were inefficient at processing *p*NPG, in part due to their high K_m_ values. For *Rg*GUS, this may be explained by the alpha helix motif adopted by Loop 1 (Fig. 2c). Docking *p*NPG into the active site reveals that the alpha helix motif would sterically clash with *p*NPG (Fig. 4c). Therefore, the propensity for the loop in *Rg*GUS to form an alpha helix may explain its low catalytic efficiency. To determine whether other L1 GUS enzymes are capable of forming an alpha helix in the active site, we aligned the Loop 1 sequences (Fig. 4d). Sequence alignment revealed that *Fp*GUS retains five of the six amino acids, termed the “NFXAA motif” where “X” is a hydrophobic residue, that make up the alpha helix conformation in the *Rg*GUS loop, indicating that *Fp*GUS may also be capable of forming this secondary structure motif even though this loop is disordered in the structure of this enzyme presented here. This structural motif may aid in rationalizing the inability of these enzymes to process small glucuronide substrates. To determine whether crystal packing causes distinct L1 conformations, we analysed the loop-based symmetry contacts of each L1 shown in Fig. 4. In *Ec*GUS, *Sa*GUS, and *Rg*GUS, crystal contacts were observed, although fewer contacts were observed for *Rg*GUS compared to *Ec*GUS and *Sa*GUS. In contrast, no crystal contacts were observed within 25 Å of the *Ee*GUS L1 structure. These findings suggest that while crystal packing may dictate the L1 conformation in some GUS enzymes, it likely plays a minor role in the formation of the L1 alpha helix motif in *Rg*GUS.

Although *Lr*GUS does not possess the NFXAA motif, we hypothesized that the EDD motif, which places three anionic side chains into the active site, precludes the entry of neutral substrates and may explain the poor *p*NPG processing activity of *Lr*GUS. The EDD motif is unique to *Lr*GUS and was not present in the six other L1 GUS enzymes characterized in the study (Figs 2c and S3b). Site-directed mutagenesis of the EDD motif to the corresponding neutral polar residues QNN led to a complete loss of GUS activity (Fig. S13b), suggesting that this negatively charged patch is critical for small glucuronide processing by *Lr*GUS.

In general, the overall trends with small molecule glucuronide substrates were maintained when comparing *p*NPG and DCF-G processing activity. However, *Ec*GUS, the fastest and most efficient GUS at cleaving *p*NPG, was the slowest at processing DCF-G. Given that that the aglycone is expected to primarily interact with the L1 in *Ec*GUS, we expect that the amino acid composition of L1 is responsible for this differential processing. Future studies will be carried out to evaluate how specific L1 residues affect *p*NPG and DCF-G processing.

### UNC10201652 is more potent than Inhibitor 1 *in vitro* against all GUS enzymes

**Figure 6:**
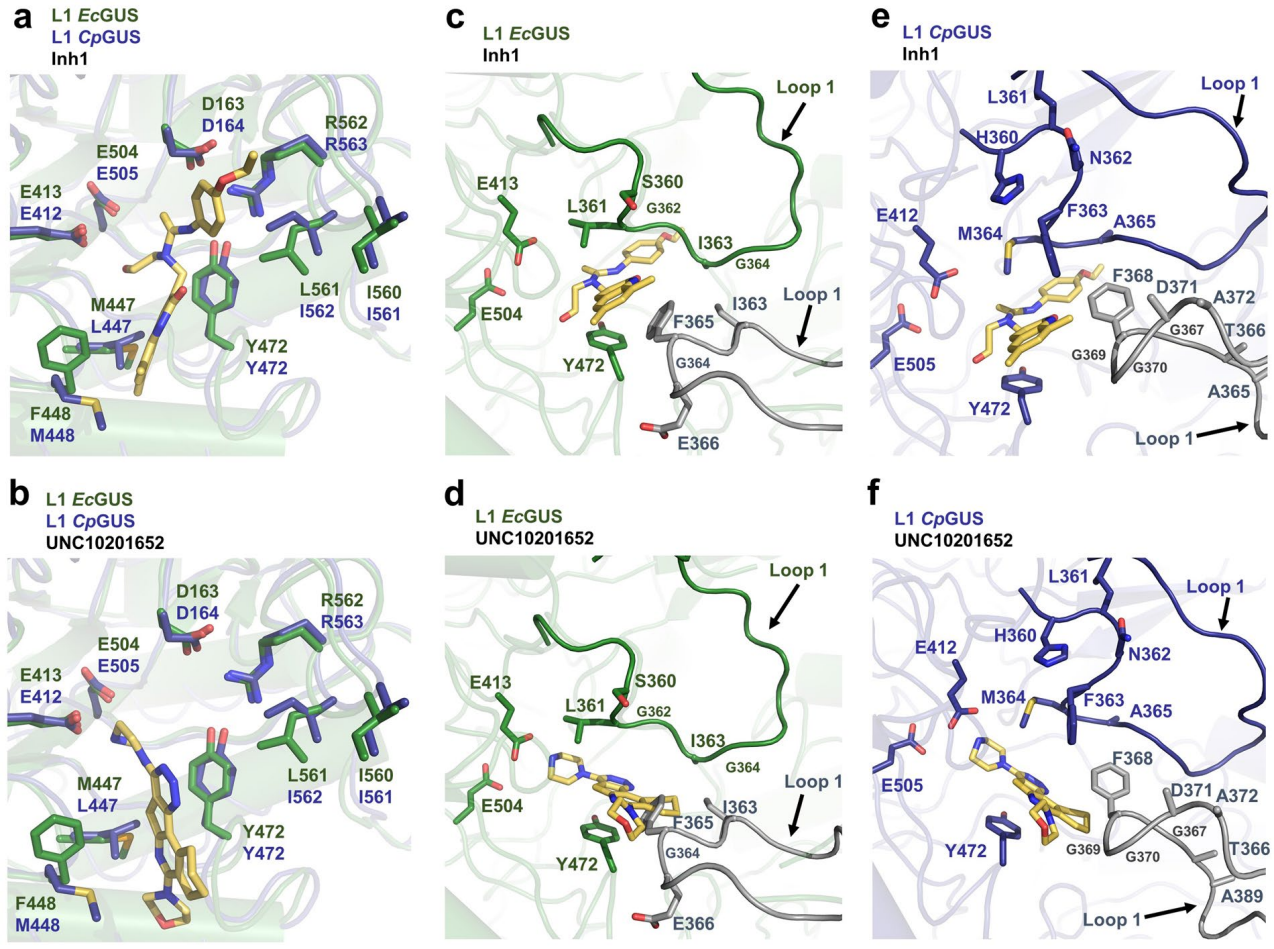
Inhibitor 1 and UNC10201652 chemotypes within the *Ec*GUS and *Cp*GUS active sites. (**a**) Overlay of *Ec*GUS and *Cp*GUS containing the Inhibitor 1 chemotype docked into the active site. Loop 1 structures are not shown. (**b**) Overlay of *Ec*GUS and *Cp*GUS containing UNC10201652 docked into the active site. Loop 1 structures are not shown. (**c**) *Ec*GUS loop 1 region with Inhibitor 1 docked into the active site. (**d**) *Ec*GUS loop 1 region with UNC10201652 docked into the active site. (**e**) *Cp*GUS loop 1 region with Inhibitor 1 docked into the active site. (**f**) *Cp*GUS loop 1 region with UNC10201652 in the active site. Loop 1 structures are indicated by arrows. Grey loops are from the adjacent monomer.

Here, we also present the IC_50_ values for Inhibitor 1 and UNC10201652 against a panel of GUS enzymes examined at two pH values. For the GUS enzymes inhibited, UNC10201652 demonstrated greater potency than Inhibitor 1 at both pH 6.5 and 7.5 (Table 2). For example, UNC10201652 was 19 times more potent against *Ec*GUS at pH 7.5 than Inhibitor 1. The mechanism of action demonstrated by UNC10201652 may provide insight into its enhanced inhibitory activity. UNC10201652 displays unique substrate-dependent and slow-binding inhibition, in which it binds to the glucuronic acid (GlcA)-enzyme catalytic intermediate as a piperazine-linked glucuronide^22^. The resulting piperazine-linked glucuronide contains a positively charged piperazine amine and is predicted to form an electrostatic interaction with the catalytic acid/base glutamate in the active site (Fig. 6b)^22^. The presence of this ionic interaction likely aids in stabilizing the bound UNC10201652-GlcA conjugate in the active site. In contrast, only hydrogen bonds and hydrophobic interactions are present in the Inhibitor 1-bound complex (Fig. 6a). In addition, a conserved tyrosine in GUS enzymes (Y472 in *Ec*GUS and *Cp*GUS) forms pi-pi stacking interactions with the UNC10201652 aromatic scaffold, which may also aid in stabilizing the inhibitor-bound complex (Fig. 6b). This tyrosine is not expected to provide pi-pi stacking interactions with Inhibitor 1 (Fig. 6a).

The formation of the UNC10201652-GlcA conjugate within the GUS active site may also explain the pH dependency of UNC10201652, in which the inhibitory activity decreases with decreasing pH (Table 2). As the pH decreases, the catalytic glutamates are expected to be protonated, which would disrupt the ionic bond between a negatively charged glutamate and the positively charged piperazine amine.

### Active site loop composition impacts L1 GUS inhibition

By comparing the IC_50_ values determined in this study, we found that the L1 Cluster GUS enzymes were not inhibited, or poorly inhibited, by the bacterial GUS inhibitors (Table 2). As stated above, the alpha helix formed in the Loop 1 of *Rg*GUS and predicted to form in the Loop 1 of *Fp*GUS may block the active site of this enzyme for productive binding to this substrate. This structural feature may also prevent Inhibitor 1 and UNC10201652 from accessing the active site pocket, resulting in the lack of inhibition observed for these L1 Cluster GUS enzymes.

Further, Inhibitor 1 and UNC10201652 displayed varying degrees of potency against the L1 GUS enzymes that were susceptible to inhibition (Table 2). The specific Loop 1 amino acid sequence likely explains the differences in inhibition observed for the L1 GUS enzymes (Fig. 4d). For example, Inhibitor 1 was found to be more potent against *Ec*GUS than *Cp*GUS (Table 2). Upon docking Inhibitor 1 into the active sites of these enzymes based on the previously determined structures of the Inhibitor 2- and Inhibitor 3-bound *Ec*GUS complexes (PDB: 3LPF and 3LPG, respectively) and the UNC10201652-bound *Cp*GUS complex, we observed that F365 present on the Loop 1 of the adjacent *Ec*GUS monomer likely serves as a primarily contact with Inhibitor 1, as previously described (Fig. 6c)^20^. In *Cp*GUS, a glycine is present at this position and may explain why Inhibitor 1 is more effective at inhibiting *Ec*GUS than *Cp*GUS (Figs 4d and 6e). In contrast, UNC10201652 was determined to be more potent towards *Cp*GUS than *Ec*GUS. Again, the specific Loop 1 composition of both enzymes may provide an explanation for this observation. Upon docking UNC10201652 into the *Ec*GUS active site, we found that F365 in the adjacent monomer may clash with the inhibitor scaffold, although the exact positioning of the loop and inhibitor is difficult to predict in the absence of a co-crystal structure (Fig. 6d). To test the role F365 plays in Inhibitor 1 and UNC10201652 potency, we determined the IC_50_ values for each inhibitor using *Ec*GUS F365 variant protein. Inhibitor 1 and UNC10201652 were less potent toward the *Ec*GUS F365 mutant than the WT (Fig. S14), suggesting that this residue aids in stabilizing both inhibitors and that the L1 in *Ec*GUS adopts a conformation that accommodates UNC10201652. Given these results, the L1 in *Cp*GUS may also adopt a conformation distinct from that displayed in Fig. 6e,f. Thus, the role specific L1 amino acids play in Inhibitor 1 and UNC10201652 binding, should be evaluated in future more expansive mutagenesis studies.

In addition, the L1 *Ee*GUS was also only moderately inhibited by UNC10201652 and not inhibited by Inhibitor 1 (Table 2). While this may be explained in part by the specific Loop 1 composition of *Ee*GUS, the presence of an additional Loop, named the “Ee Extra Loop”, distinct from the Loop 1 or Loop 2 position may also impede inhibitor binding in the active site (Figs 4b and S3a). To determine whether the Ee Extra Loop affects inhibitor potency, we deleted residues M149 to A159, which comprise the loop. Deletion of this region led to a significant loss of *p*NPG activity, with the mutant exhibiting 9% of the cleavage rate demonstrated by the WT (Fig. S13c). Circular dichroism (CD) analysis revealed that deletion of the Ee Extra Loop did not alter the overall fold of the enzyme (Fig. S15a). Thus, this additional loop appears to be important in small glucuronide processing.

### C-terminal domains affect substrate processing and GUS inhibition

The crystal structure of NL *Bd*GUS presented here, as well as the previously determined structures of non-L1 GUS enzymes, revealed the presence of additional C-terminal domains, including carbohydrate binding modules (CBMs) and domains of unknown function (DUFs). These additional domains may also provide additional rationale as to why non-L1 GUS enzymes, in general, process *p*NPG less efficiently than L1 GUS enzymes, which do not possess CBMs or DUFs (Figs 3a, S1, S16 and Table 1). The C-terminal domains cause non-L1 GUS enzymes to adopt more open active sites, as opposed to the “C-term-mediated” tetrameric state exhibited by L1 GUS enzymes that create smaller, more intimate active sites (Figs 2b and 3b). Such a configuration favours larger glucuronide-containing polysaccharide substrates over smaller drug glucuronides. In addition, CBMs, like that found in *Bd*GUS, have been reported to mediate the binding and positioning of large carbohydrate substrates into the active site^29^, and their presence may indicate that larger polysaccharides are the native substrate of the GUS enzymes.

As noted above, *Bd*GUS and *Bu*GUS share highly similar tertiary and quaternary structures, but the catalytic efficiency of *Bd*GUS was nearly 10-fold less than that of *Bu*GUS (Fig. S4 and Table 1). Given that the main structural difference between these two enzymes is the presence of a CBM loop insert in *Bd*GUS, the CBM loop may explain this difference in activity, as it may occlude a substrate like *p*NPG from being properly positioned in the active site. Such considerations address the binding of substrate to these enzymes (K_m_). It is also noteworthy that the k_cat_ of the two enzymes also differ by 3-fold, something that we cannot explain using static structures and may involve distinctions in active site motion, as outlined elegantly for other enzyme systems^30^.

**Figure 7:**
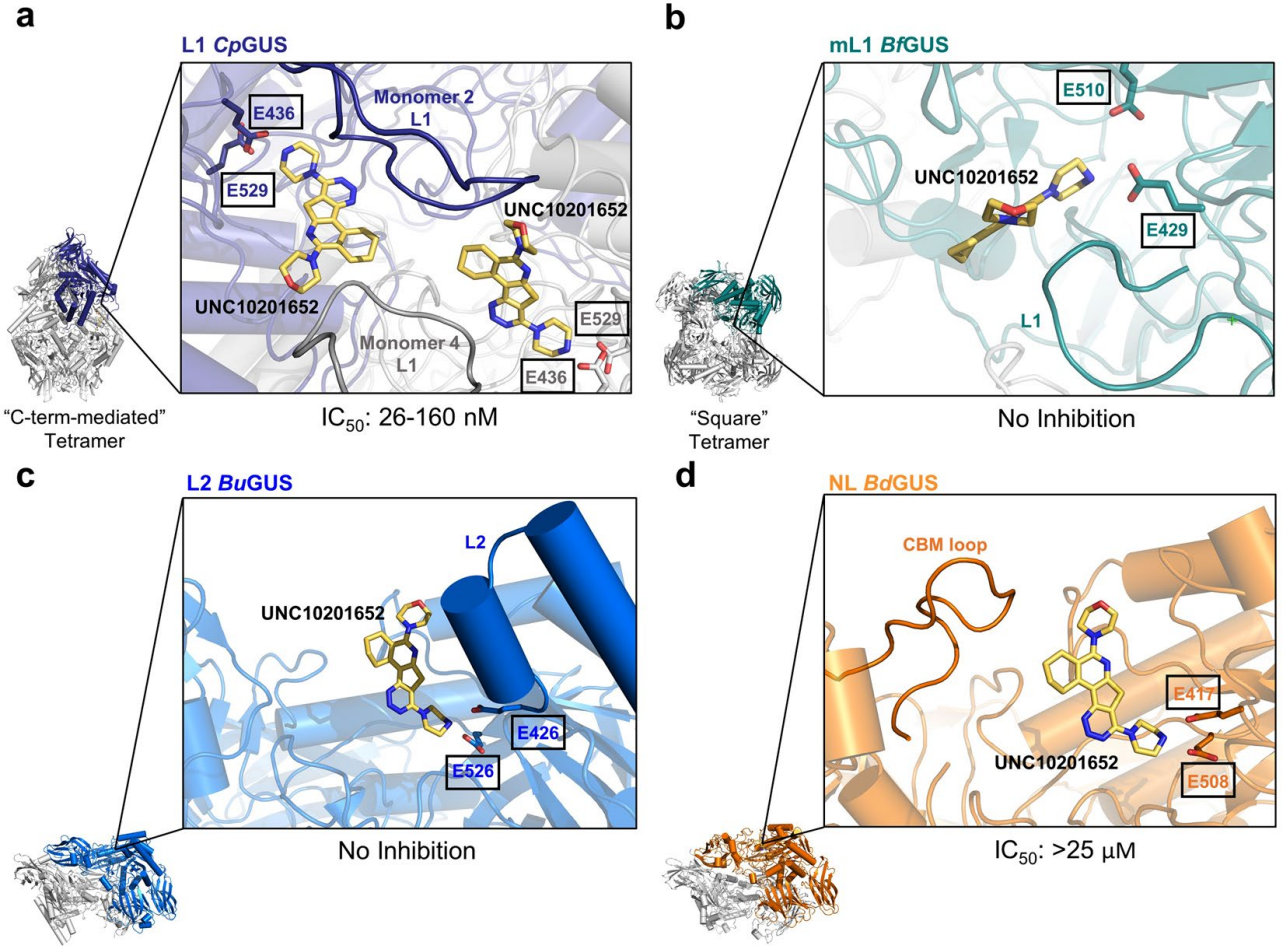
Inhibitor positioning within the GUS active site in relation to the distinct quaternary assemblies of GUS proteins. (**a**) *Cp*GUS active site shown within its “C-term-mediated” tetrameric state and Loop 1 regions from adjacent monomers contacting UNC10201652. (**b**) *Bf*GUS active site shown within its “square” tetrameric state and no loops from adjacent monomers entering the active site. (**c**) *Bu*GUS active site shown within its dimeric state. (**d**) *Bd*GUS active site shown within its dimeric state and the possibility of contacts with its CBM loop. UNC10201652 is docked into each active site based on the crystal structure of *Cp*GUS in complex with UNC10201652.

In addition, the presence or absence of C-terminal domains likely affects the binding of our GUS inhibitors. Here, we show that, with the exception of *Bd*GUS, only GUS enzymes that do not contain a C-terminal domain are inhibited. The previously reported inhibitor-bound structures of *Ec*GUS reveal that the selective GUS inhibitors make contacts with both the Loop 1 of the primary monomer as well as the Loop 1 of the adjacent monomer in its functional “C-term-mediated” tetrameric state (Fig. 7a)^17,21^. However, the presence of extra domains at the C-terminus of the GUS protein causes the enzyme to adopt either a “square” tetrameric state (e.g. *Bf*GUS) or dimeric states (e.g. *Bu*GUS and *Bd*GUS) (Fig. 7b–d). These quaternary assemblies preclude the positioning of an additional loop near the active site of the primary monomer, resulting in fewer putative inhibitor contacts. Therefore, the lack of a “C-term-mediated” tetrameric state due to the presence of one or more C-terminal domains presents a plausible explanation as to why no inhibition was observed in the majority of non-L1 GUS enzymes. *Bd*GUS, the exception to this observation, was slightly inhibited by UNC10201652. However, the CBM loop in *Bd*GUS may enter into the *Bd*GUS active site and provide stabilizing contacts, enabling slight inhibition (Fig. 7d). To evaluate the role the CBM loop plays in UNC10201652 inhibition, we deleted residues N737 to Y499 that form the CBM loop in *Bd*GUS. While deletion of this region did not alter the stability of the *Bd*GUS mutant, as assessed by CD-monitored thermal denaturation, it rendered the enzyme inactive with *p*NPG, preventing further inhibition studies (Figs S13a and S15d).

### Concluding remarks

In summary, this work provides insight into the structural and functional diversity among GUS enzymes, specifically focusing on those of the L1 class, and advances our knowledge of the specific GUS enzymes that are capable of reactivating drug glucuronides in the gut. We have shown that not all L1 GUS enzymes efficiently process small glucuronide substrates and have provided several structural explanations to support these observations. However, those enzymes that process small substrate glucuronides well are inhibited by UNC10201652.

## Methods

### Uniprot protein accession codes

The Uniprot accession codes for the proteins examined here are: *Ec*GUS (P05804), *Ee*GUS (C4Z6Z2), *Sa*GUS (Q8E0N2), *Cp*GUS (Q8VNV4), *Lr*GUS (C2JTS9), *Rg*GUS (R5TSA0), *Fp*GUS (C7H4D2), *Bf*GUS (Q5LIC7), *Bd*GUS (C3R9X4), *Bu*GUS (A0A078SUX9), *Bo*GUS (A7LZ25), and *Pm*GUS (A7AG62).

### SSN construction

The sequence similarity network diagram of GUS enzyme sequences was generated using the online enzyme function initiative-enzyme similarity tool (EFI-EST)^31^. The sequences obtained from the GUS rubric were used in combination with the EFI-EST “fasta” tool to create a sequence with 282 nodes. Each node represents sequences bearing ≥90% sequence identity to each other. A BLAST E-value of 1 × 10^−220^ was employed.

### Enzyme cloning

The GUS genes from *Lactobacillus rhamnosus* and *Ruminococcus gnavus* were purchased from Bio Basic in the pUC57 vector. The genes were amplified by PCR and inserted into the ligation independent cloning vector (LIC) pLIC-His using the primers shown in Table S2. The pLIC-His vector contains a N-terminal 6× -histidine tag. For *Lr*GUS and *Fp*GUS crystallisation, an additional 36 amino acids and 33 amino acids were added to the C-termini of the *Lr*GUS and *Fp*GUS proteins, respectively, using the C-term primers (Table S2).

### Site-directed mutagenesis

The *Ee*GUS (−)EeLoop, *Ec*GUS F365A, *Lr*GUS EDD to QNN, and *Bd*GUS (−)CBM Loop mutants were created using site-directed mutagenesis. Primers were synthesized by Integrated DNA Technologies and are shown in Table S2. The mutant plasmids were sequenced to confirm the mutations. The mutants were produced and purified using *E. coli* BL21 (DE3) Gold as described below.

### CD analysis of GUS mutants

The protein stabilities of the WT and mutant GUS enzymes described above were determined using the Circular Dichroism method^31^. Enzyme (2.5 µM) in CD buffer containing 10 mM potassium phosphate (pH 7.4) and 100 mM potassium fluoride was loaded into a 1-mm pathlength cuvette. Using a Chirascan-plus instrument (Applied Photophysis Limited), spectra from 185 to 260 nM were recorded at 20 ± 1.0 °C. Measurements were corrected for background signal using a CD buffer sample. The melting profile of the sample (2.5 µM) was monitored at 228, 220, 225, and 218 nm for *Ec*GUS, *Ee*GUS, *Lr*GUS, and *Bd*GUS, respectively, from 20 °C to 94 °C.

### Enzyme expression and purification

All GUS enzymes were expressed and purified as previously described^20^. Expression and purification of *Lr*GUS and *Rg*GUS was identical to that previously described for *Ee*GUS^20^. Briefly, *Lr*GUS and *Rg*GUS expression plasmids were transformed into BL21 DE3 Gold cells. Cells were grown in the presence of ampicillin in LB at 37 °C until the OD_600_ reached 0.5, at which point the temperature was reduced to 18 °C. At OD_600_ ~0.8, protein expression was induced by the addition of 0.1 mM isopropyl-1-thio-D-galactopyranoside (IPTG), and cells were incubated overnight. Cells were pelleted by centrifugation at 4500 × g for 20 min at 4 °C. Cell pellets were resuspended in Buffer A (20 mM potassium phosphate pH 7.4, 50 mM imidazole, 500 mM NaCl), DNase, lysozyme, and a Roche complete EDTA free protease inhibitor tablet. Proteins contained 6x histidine tags and were purified by Ni-column affinity chromatography and eluted with Buffer B (20 mM potassium phosphate pH 7.4, 500 mM imidazole, 500 mM NaCl). After Ni-column chromatography, the proteins were subjected to size-column chromatography using Buffer C (20 mM HEPES pH 7.4, 50 mM NaCl). Protein eluents were then concentrated, flash frozen in liquid nitrogen, and stored at −80 °C.

### *In vitro* pNPG processing assay

The standard substrate *p*-nitrophenyl-β-D-glucuronide (*p*NPG) was purchased as a solid (Sigma Aldrich) and resuspended in water to a concentration of 100 mM. *In vitro* assays were conducted in 96-well, clear bottom assay plates (Costar, Tewksbury MA) at 37 °C in a 50 µL total volume. Reactions consisted of 10 µL assay buffer (50 mM HEPES, 50 mM NaCl, various pH), 10 µL enzyme (various concentrations), and 30 µL *p*NPG (various concentrations) diluted in assay buffer. Product formation was measured at 410 nm using a PHERAstar Plus Microplate reader (BMG Labtech). To determine the optimal pH for *Sa*GUS, *Cp*GUS, *Lr*GUS, and *Rg*GUS, the above assay was conducted at various enzyme concentrations and 800 µM *p*NPG in the appropriate assay buffer where the pH ranged from 4.0 to 7.4. For assays at pH 6.0 or lower, reactions were quenched with 100 µL of 0.2 M sodium carbonate, and product formation was measured over time via absorbance at 410 nm. For reactions at pH 6.5 or above, reactions were not quenched. For *Cp*GUS and *Rg*GUS, pH 6.5 was selected as the optimal pH due to the ability to monitor product formation continuously at this pH. Upon determining the optimal pH for each enzyme, velocities were determined for multiple substrate and enzyme concentrations at each enzyme’s optimal pH, and the Michaelis-Menten kinetics module in SigmaPlot was used to calculate K_m_, k_cat_, and catalytic efficiency.

*Rg*GUS and *Bo*GUS displayed complex substrate inhibition kinetics, and we were not able to fit these to established substrate inhibition models, including the uncompetitive substrate inhibition model, as determined in Equation 1, nor by the substrate inhibition models outlined in Lin *et al*. or Yoshino *et al*.^26,27^.

1 $${\rm{v}}=\frac{{{\rm{V}}}_{{\rm{\max }}}[{\rm{S}}]}{{{\rm{K}}}_{{\rm{M}}}+[{\rm{S}}](1+\frac{[{\rm{S}}]}{{{\rm{K}}}_{{\rm{i}}}})}$$

Therefore, apparent k_cat_ and K_m_ values for *Rg*GUS and *Bo*GUS were estimated by plotting velocity versus *p*NPG concentrations at which no inhibition was observed.

### *In vitro* DCF-G processing assay

Diclofenac acyl β-D-glucuronide (DCF-G) was purchased as a solid (LC Scientific Inc., Concord ON) and resuspended in DMSO to a concentration of 25 mM. *In vitro* assays were conducted at 37 °C in a 50 µL total volume. Reactions consisted of 10 µL assay buffer (50 mM HEPES, 50 mM NaCl, various pH), 10 µL enzyme (various concentrations), and 30 µL DCF-G (400 µM final) diluted in assay buffer. The pH of each reaction was chosen based on the optimal pH determined for each GUS with *p*NPG. Reactions were quenched at 0, 1, 2, 3, 4, and 5 min with 50 µL of 25% trichloroacetic acid (TCA). For *Pm*GUS, reactions were quenched at 0, 10, 20, 30, 40, and 50 min. After centrifugation at 13,000xg for 10 min, the resultant supernatant was subjected to UPLC analysis. The concentration of DCF-G remaining at each time point was quantified on a Waters Acquity H-class liquid chromatograph system. Samples were separated on a Waters Acquity UPLC BEH C18 column (2.1 × 50 mm, 1.7 µm particle size) at 40 °C. The flow rate was 0.6 mL/min, and the injection volume was 3 µL. LC conditions were set at 100% water with 0.1% formic acid (A) ramped linearly over 9.8 mins to 95% acetonitrile with 0.1% formic acid (B) and held until 10.2 mins. At 10.21 mins the gradient was switched back to 100% A and allowed to re-equilibrate until 11.25 mins. DCF-G was monitored at 280 nm. The concentration of DCF-G was determined from a standard curve (0–500 µM DCF-G in assay buffer). Control reactions were performed in which enzyme was substituted with buffer. Background hydrolysis was not observed at each pH tested. Reactions were performed in triplicate for each enzyme.

### pNPG processing assay in faecal extracts

All animal studies were approved by the University of North Carolina Institutional Animal Care and Use Committee (IACUC), in accordance with the Care and Use of Laboratory Animals guidelines set by the National Institutes of Health. Twelve-week old female C57/BL6J mice were individually housed in specific pathogen-free conditions with sterile ventilator cages containing corn bedding, with *ad libitum* access to chow and water. Prior to treatment, faecal pellets were collected from each mouse shortly by gentle abdominal palpation and snap frozen in sterile microfuge tubes. Animals received a single ulcerogenic dose of DCF (60 mg/kg) by intraperitoneal injection, as previously described^18^. Twelve hours following DCF exposure, another set of faecal pellets were collected and stored as described above. To perform the assay, frozen faecal samples were rehydrated in 15× assay buffer (weight/volume; 20 mM HEPES, 50 mM NaCl, pH 7.4, 1 × Complete® Protease inhibitor cocktail (Roche). Bacterial cells were lysed using a Tissuelyzer II (Qiagen) for 2 min at 30 Hertz. Homogenate was sonicated for 4 min, and then clarified by centrifugation for five minutes at 13,000×g. All experimental manipulation until this point occurred at 4 °C. 5 μL of faecal slurry supernatant was used to initiate the hydrolysis reaction of 1 mM pNPG resuspended in the same buffer. Parallel reactions containing only pNPG or only buffer/faecal slurry were used as negative controls; an aliquot of each sample was heat inactivated at 95 °C and used in the assay for further background establishment. Each sample was assayed using three technical replicates. The initial velocities of the resultant progress curves of the reaction were calculated in MATLAB by linear regression, and then normalized to the total faecal protein content calculated using a standard Bradford assay.

### *In vitro* inhibition assay

*In vitro* inhibition of bacterial GUS enzymes by UNC10201652 was assessed as previously described^22^. Reactions consisted of 5 µL of GUS (15 nM final for *Ec*GUS, *Ee*GUS, *Sa*GUS, and *Cp*GUS; 150 nM final for *Rg*GUS, *Lr*GUS, and *Bd*GUS), 5 µL of inhibitor (various concentrations), 30 µL of *p*NPG (900 µM final), and 10 µL of assay buffer (25 mM NaCl, 25 mM HEPES, pH 6.5 or pH 7.5 final). Reactions were initiated by addition of *p*NPG and then incubated for 1 hour, after which the end point absorbance was determined. Due to the slow-binding nature of UNC10201652, the IC_50_ was determined as the inhibitor concentration that yielded a 50% reduction in the maximum absorbance of the uninhibited reaction, where percent inhibition was calculated as:

$$ \% \,{\rm{inhibition}}=[1-(\frac{{{\rm{A}}}_{\exp }-{{\rm{A}}}_{{\rm{bg}}}}{{{\rm{A}}}_{{\rm{\max }}}-{{\rm{A}}}_{{\rm{bg}}}})]\times 100$$

where *A*_*exp*_ is the end point absorbance at a particular inhibitor concentration, *A*_*max*_ is the absorbance of the uninhibited reaction, and *A*_*bg*_ is the background absorbance the assay. Percent inhibition values were subsequently plotted against the log of inhibitor concentration and fit with a four-parameter logistic function in SigmaPlot 13.0 to determine the IC_50_.

*In vitro* inhibition of bacterial GUS enzymes by Inhibitor 1 was determined using the reaction conditions described above, but the IC_50_ was determined as the inhibitor concentration that yielded 50% reduction in the maximum initial velocity of the uninhibited reaction.

### Crystallization and structure determination

Crystals of *Lr*GUS, *Rg*GUS, *Bd*GUS and *Fp*GUS were produced via the hanging-drop vapor diffusion method. *Lr*GUS crystals were formed by incubation of 5 mg/mL *Lr*GUS in 17% PEG3350, 0.4 M NaCl, and 0.1 Tris HCl pH 7.4 at 20 °C. *Rg*GUS crystals were formed by incubation of 16 mg/mL *Rg*GUS in 1 M diammonium hydrogen citrate and 0.05 M sodium acetate pH 4.5 at room temperature. *Bd*GUS crystals were formed by incubation of 11.2 mg/mL *Bd*GUS in 16% PEG3350 and 0.35 M sodium citrate at 20 °C. *Fp*GUS crystals were formed by incubation of 13 mg/mL *Fp*GUS in 0.1 M bis-tris propane: HCl, pH 7.0 and 0.4 M magnesium formate at 20 °C. For all crystals, 15% glycerol was used as the cryoprotectant. Diffraction data for all crystals were collected at APS beamline 23-ID-D and ID-B, and data were collected at 100 K. *Lr*GUS, *Rg*GUS, and *Fp*GUS were solved via molecular replacement with *E. coli* GUS (PDB: 3LPG) in the software Phenix. *Bd*GUS was solved via molecular replacement with *Bacteroides uniformis* GUS (PDB: 5UJ6). Refinement was also performed in Phenix, with initial rounds of simulated annealing. Final coordinates and structure factors have been submitted to the RCSB and assigned accession codes 6ECA, 6EC6, 6ED2, and 6ED1 for the *Lr*GUS, *Rg*GUS, *Fp*GUS, and *Bd*GUS structures, respectively.

### SEC-MALS analysis of GUS enzymes

*Rg*GUS, *Lr*GUS, *Fp*GUS, and *Bd*GUS were analysed on a Superdex 200 size exclusion column connected to an Agilent FPLC system, Wyatt DAWN HELEOS II multi-angle light scattering instrument and a Trex refractometer. The injection volume was 50 μL. *Rg*GUS, *Lr*GUS, and *Fp*GUS were assessed at 3 mg/mL, and *Bd*GUS was assessed at 10 mg/mL in 50 mM HEPES and 150 mM NaCl pH 7.4 buffer. A flow rate of 0.5 mL/min was used. Light scattering and refractive index data were collected and analysed using Wyatt ASTRA (Ver. 6.1) software. A dn/dc value of 0.185 was used for calculations, and a band-broadening correction was applied. Almost 100% of *Rg*GUS, *Lr*GUS, and *Fp*GUS eluted in single peaks with weight-average molar masses of 291.5 kDa, 284.2 kDa, and 293.0 kDa, respectively, indicating that they form stable tetramers in solution. In contrast, *Bd*GUS eluted in a single peak with a small trailing edge with a weight-average molar mass of 206.3 kDa, indicating that it is in dimer-tetramer equilibrium with dimer being the predominant species.

## Electronic supplementary material

Supplementary Info

## Data Availability

The data sets generated during and/or analysed are either included in the published article or available from the corresponding author on reasonable request.
