# Supplementary material for: Structure, function, and inhibition of drug reactivating human gut microbial β-glucuronidases
Source: Sci Rep. 2019 Jan 29;9:825. doi: 10.1038/s41598-018-36069-w (PMC6351562; doi:10.1038/s41598-018-36069-w)
Supplement: Supplementary file 1 — Supplementary Info [file 41598_2018_36069_MOESM1_ESM.pdf]

Structure, function, and inhibition of drug reactivating human gut microbial β-glucuronidases

Kristen A. Biernat,[1] Samuel J. Pellock,[1] Aadra P. Bhatt,[2] Marissa M. Bivins,[3] William G. Walton,[1] Bich Ngoc T. Tran,[1] Lianjie Wei,[1] Michael C. Snider,[1] Andrew P. Cesmat,[1] Ashutosh Tripathy,[4] Dorothy A. Erie,[1] and Matthew R. Redinbo[1,4,5,6*]

[1]Department of Chemistry, University of North Carolina at Chapel Hill, Chapel Hill, NC 27599, USA; [2]Department of Medicine, University of North Carolina at Chapel Hill, Chapel Hill, NC 27599, USA; [3]Department of Pharmacology, University of North Carolina at Chapel Hill, Chapel Hill, NC 27599, USA; [4]Department of Biochemistry and Biophysics, University of North Carolina at Chapel Hill, Chapel Hill, NC 27599, USA; [5]Department of Microbiology and Immunology, and Integrative Program for Biological and Genome Sciences, University of North Carolina at Chapel Hill; Chapel Hill, NC, 27599, USA; [6]Lead contact

[*]Correspondence: redinbo@unc.edu

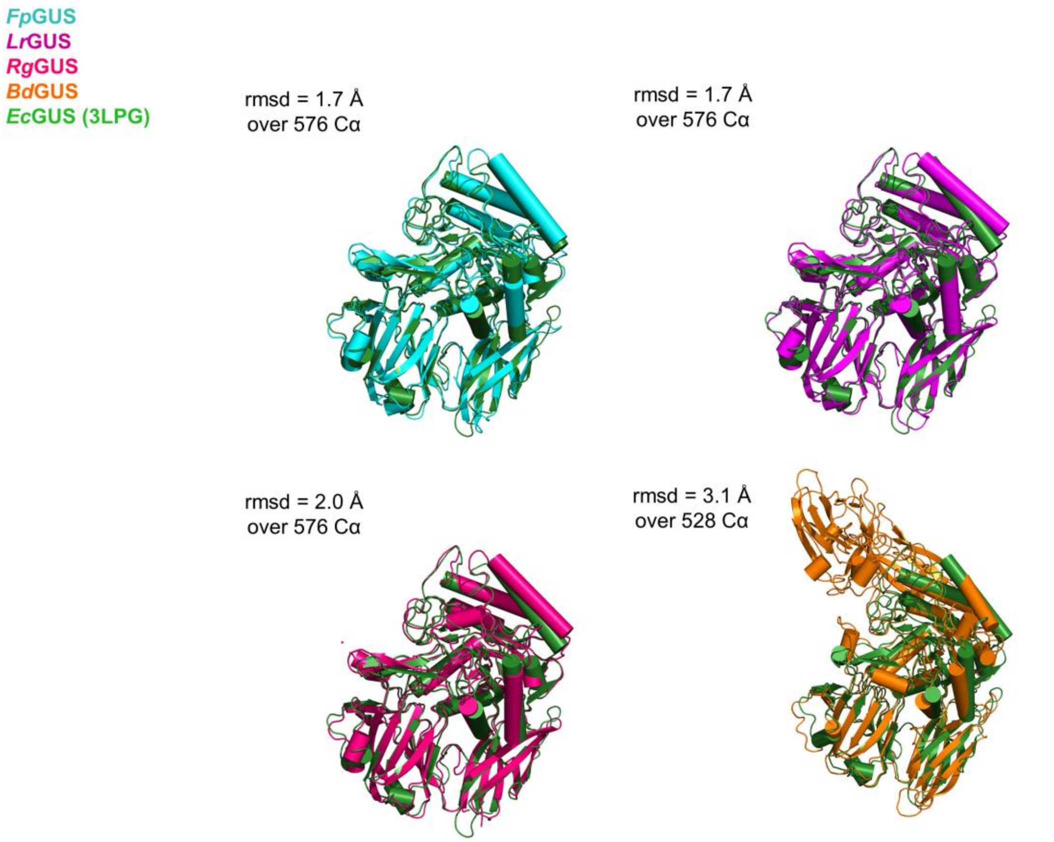

Figure S1. Structural alignments of the monomers of *Fp*GUS (*cyan*), *Lr*GUS (*magenta*), *Rg*GUS (*dark pink*), and *Bd*GUS (*orange*) with the previously elucidated structure of *Ec*GUS (dark green, PDB: 3LPG).

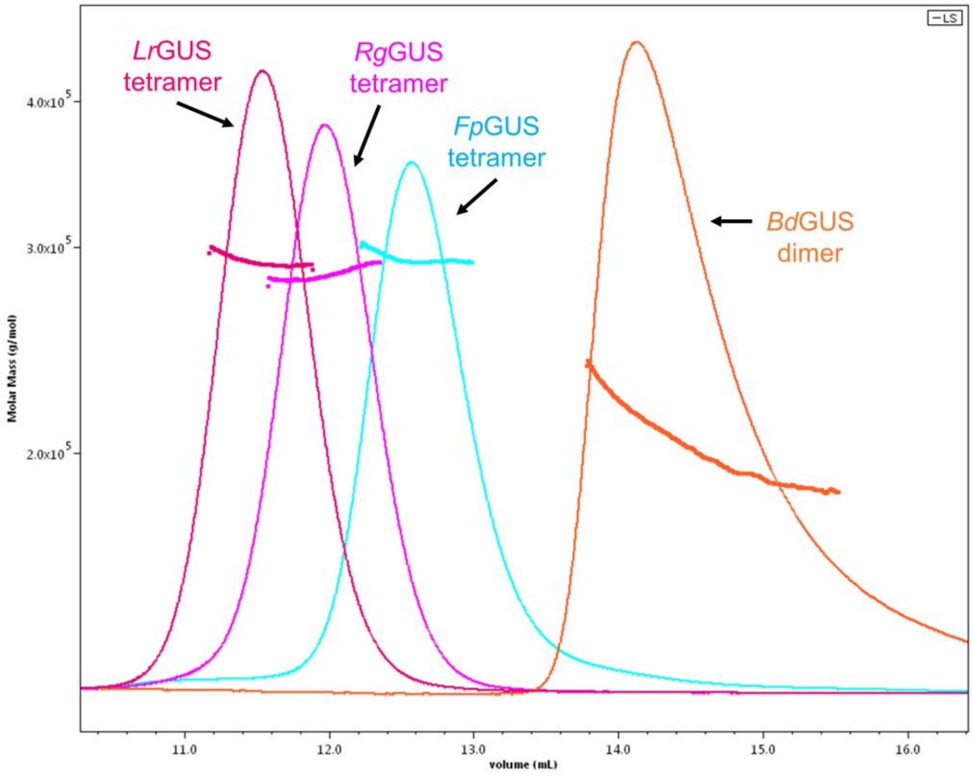

Figure S2. Size-exclusion chromatography multi-angle light scattering analysis (SEC-MALS) of *Lr*GUS, *Rg*GUS, *Fp*GUS, and *Bd*GUS confirms oligomeric states predicted from the crystal structures.

a

Ee Extra Loop

```
EcGUS      ADVTPYVIAGKSVRITVCVNNELNWQTIPPGMVIT-----------DENGKKKQSYFHDF      164
EeGUS      VELNDDLQDG-DNLLTIAVNNVIDYTTLPVGGKANMMSGMMGGMGAGASDKPQNNPNFDF      173
SaGUS      VLVPECKYNNEKIKVSICANNVLDYTTLPVGNYSEII-------QEDGSIKKKVRENFDF      161
CpGUS      AEINDLLVSG-DNRLTVAVNNIIDETTLPVGLVKEV--------EVDGKKVIKNSVNFDF      165
FpGUS      ATVTNIVRYNQFNKLSVLANNELSETMLPAGTTCTL-----------ADGRKIAAPYFDF      167
LrGUS      ADISEAVKYGAENTVVVKGNNELSREALPAGDTITL-----------RNGKKMVRPFFDF      167
RgGUS      ADISTVAKPGQVNQVVVKINNELNETSLPCGATKIL-----------NNGRKLAKPYFDF      168
           . :      .    : :  ** :.   :* *                 .        .**
```

b

```
EcGUS      IKELIARDKNHPSVVMWSIANEPDTRPQGAREYFAPLAEATRKLDPT-RPITCVNVMFCD      450
EeGUS      IRDLISRDKNHACVVMWSIANEPDSAAEGAYDYFKPLYDLARELDPQKRPCTLVSVQGTT      463
SaGUS      IQELVKRDKNHPSVVMWVVANEPASHEAGAHDYFEPLVKLYKDLDPQKRPVTLVNILMAT      446
CpGUS      LRELVSRDKNHPCVVMWSVANEPDSDSEGAKEYFEPLIKLTKELDPQKRPVTVVTYLMST      450
FpGUS      LTDMIDRDKNHPSVIAWSLLNEPQCTSAGTEEYFKPLFELARRLDPQKRPRTYTVLMTSL      456
LrGUS      IRDLIKRDKNHPSVLAWSLFNEPDTIDENAVPYFKQIFDESKDLDPQGRPRTFTLSEDDT      453
RgGUS      TEEMITRDKNHPSVIAWSLFNEPETITDYAYEYFKEVFAAAETYDFQSRPMTGAFEKNSK      456
             ::: ***** .*: * : ***      :  **  :    .  *   ** * .
```

EDD motif

Figure S3. Sequence alignments of selected regions in L1 GUS enzymes. (**a**) L1 GUS alignment showing the extra active site loop in *Ee*GUS highlighted in magenta. (**b**) L1 GUS alignment showing the EDD motif unique to *Lr*GUS in blue.

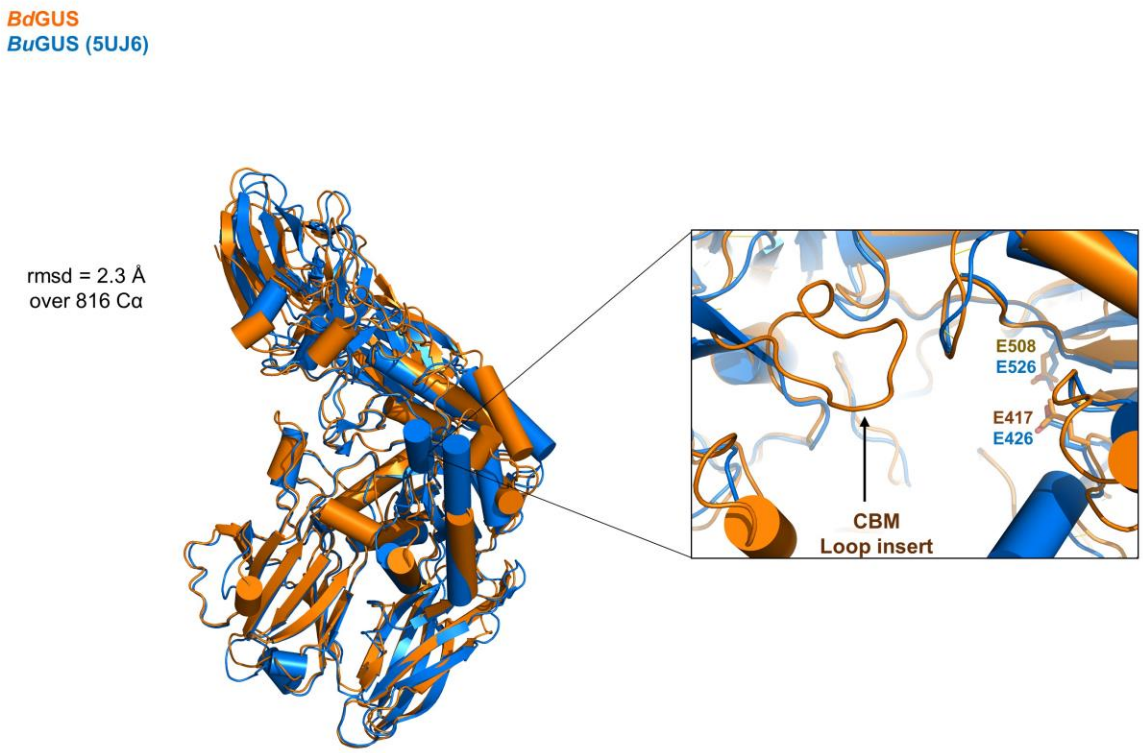

Figure S4. Structural alignment of *Bd*GUS (*orange*) and *Bu*GUS (*blue*).

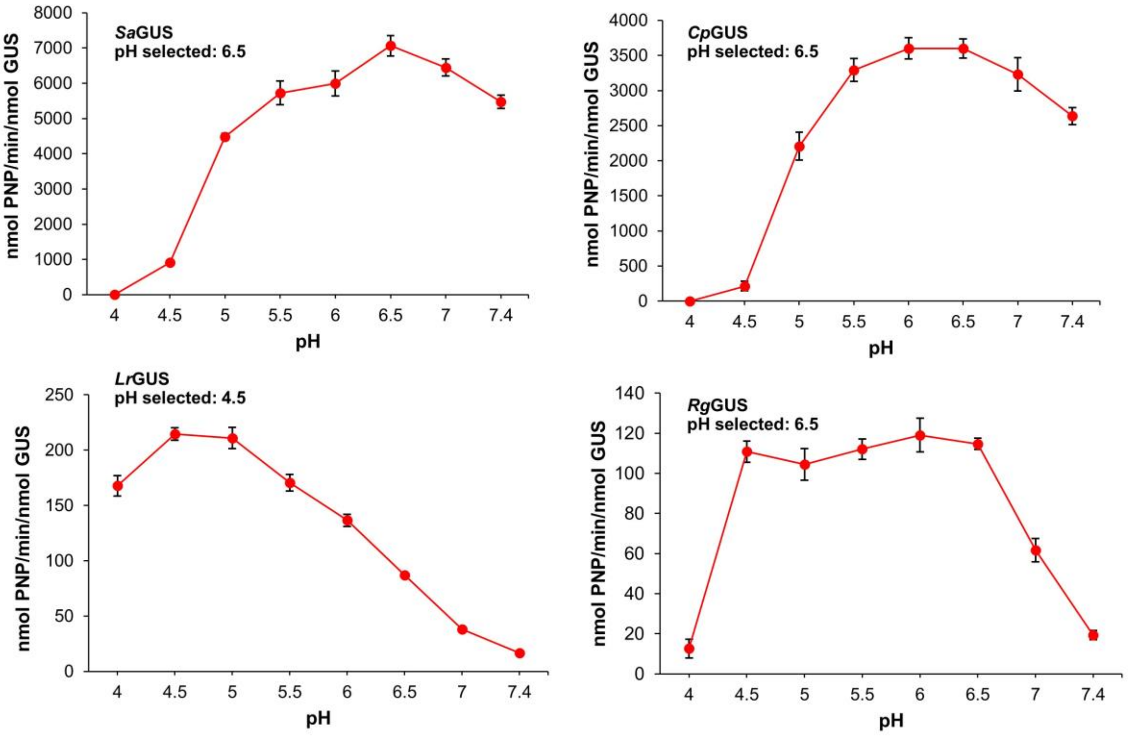

Figure S5. pH screen of *p*NPG hydrolysis for *Sa*GUS, *Cp*GUS, *Lr*GUS, and *Rg*GUS. Error bars represent SEM of n=3 biological replicates.

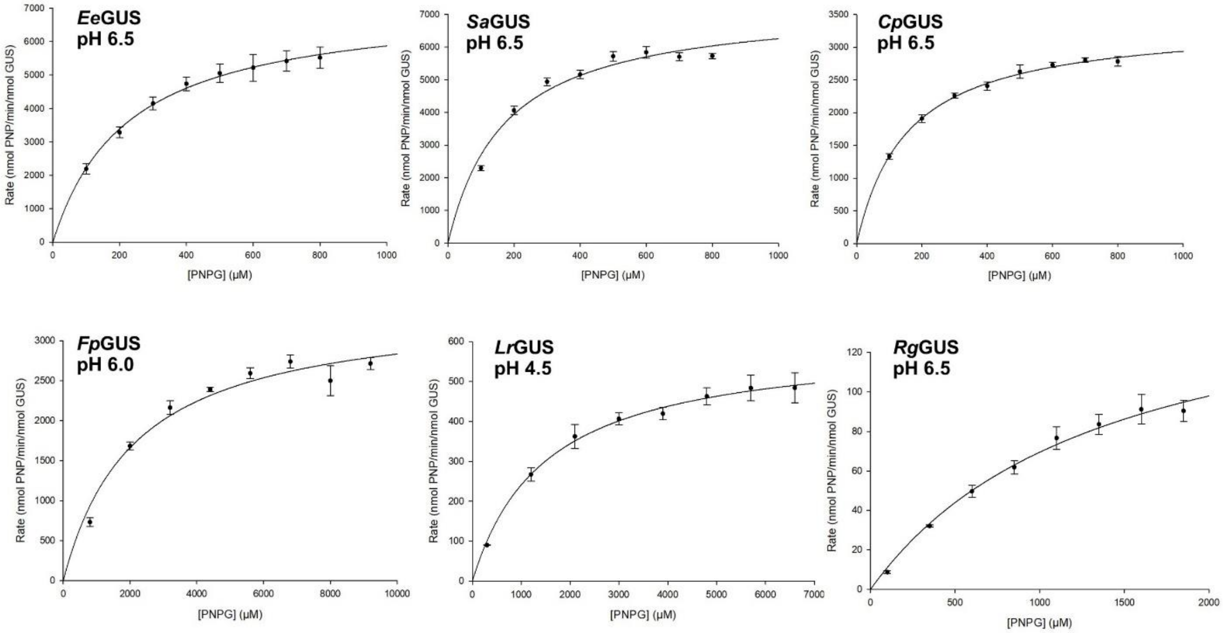

Figure S6. Michaelis Menten curves of L1 GUS enzymes with *p*NPG at optimal pH.

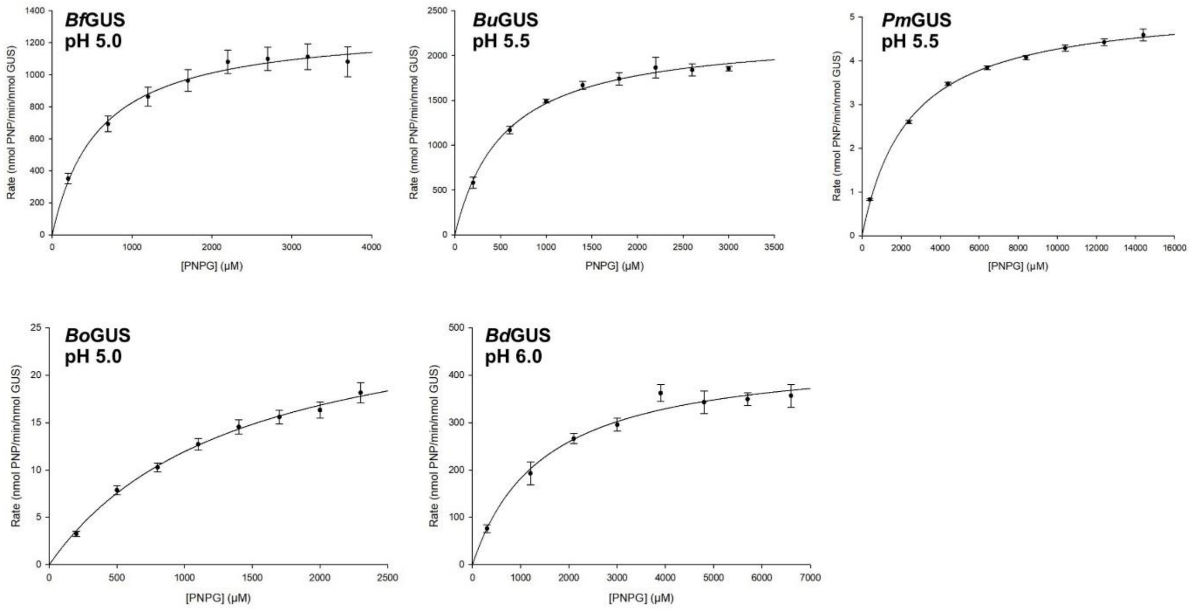

Figure S7. Michaelis Menten curves of non-L1 GUS enzymes with *p*NPG at optimal pH.

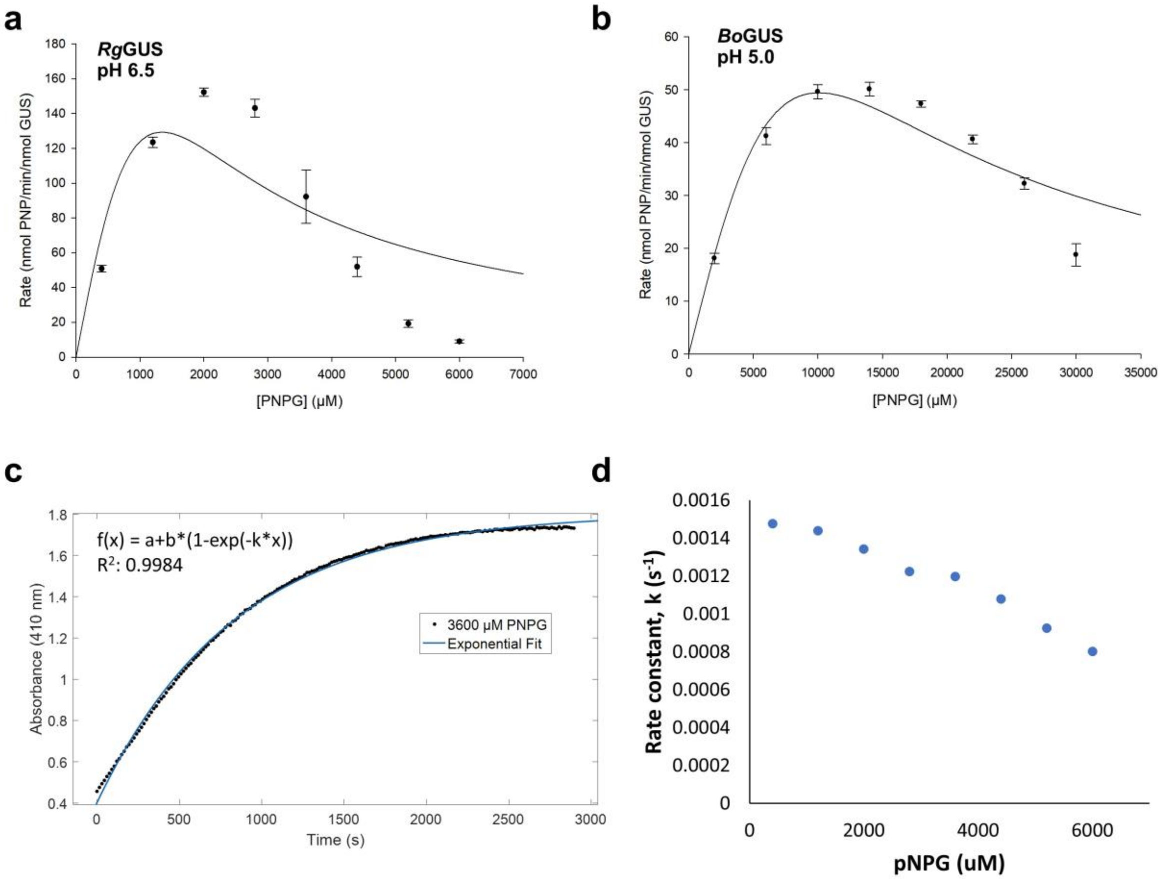

Figure S8. Substrate inhibition of *Rg*GUS and *Bo*GUS catalyzed hydrolysis of *p*NPG. (**a**) *Rg*GUS velocity as a function of *p*NPG concentration. The uncompetitive substrate inhibition model does not appear to fit the data. (**b**) *Bo*GUS velocity as a function of *p*NPG concentration. The uncompetitive substrate inhibition model does not appear to fit the data. (**c**) Example progress curve for *Rg*GUS at 3600 µM *p*NPG. The progress curves demonstrate single exponential behavior, indicating a lack of product inhibition. (**d**) Plot of rate constant versus *p*NPG concentration for *Rg*GUS. Decreasing rate constants with increasing *p*NPG concentrations are indicative of substrate inhibition.

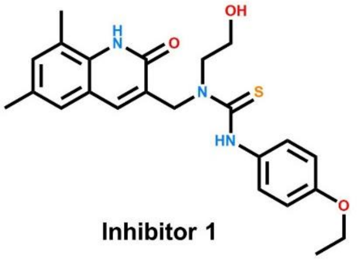

Inhibitor 1

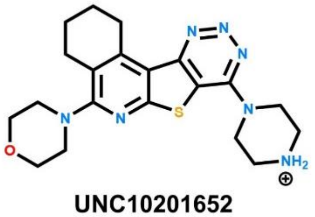

UNC10201652

Figure S9. Chemical structures of Inhibitor 1 and UNC10201652.

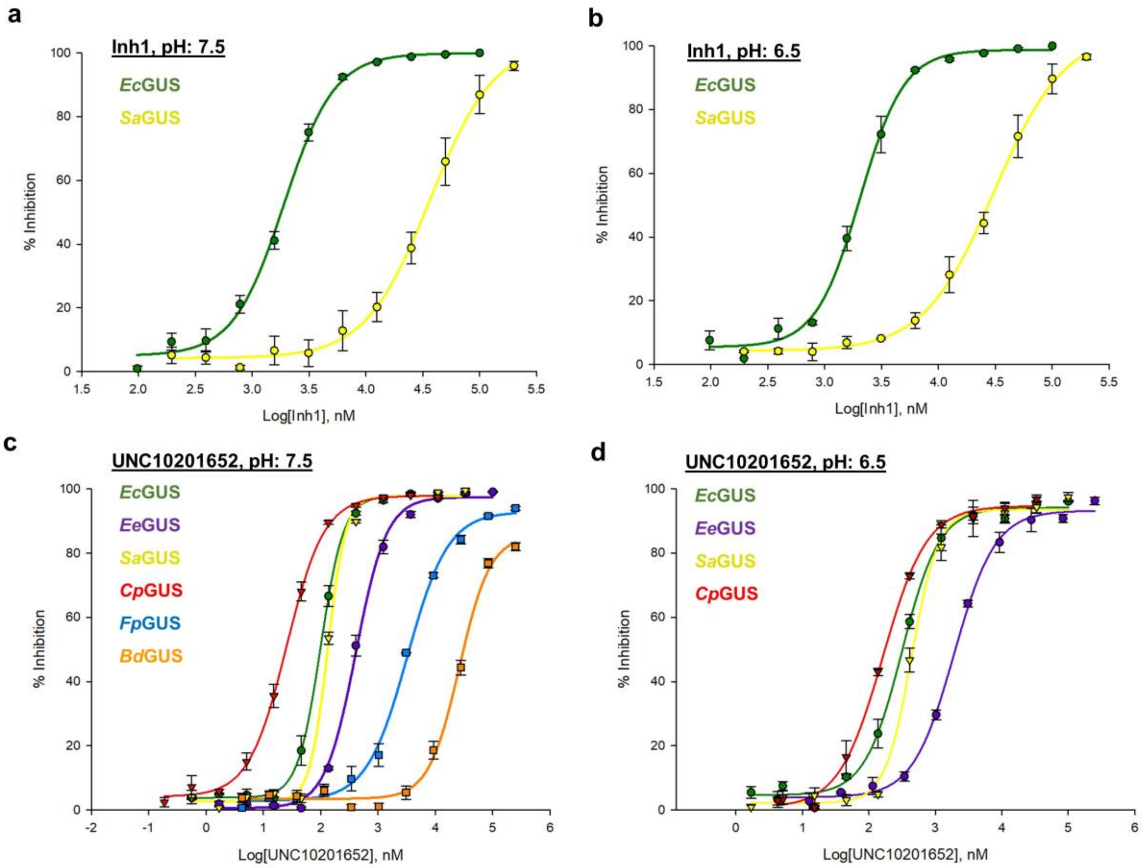

Figure S10. Inhibition of GUS enzymes by GUS-specific inhibitors. (**a**) IC_{50} curves of Inhibitor 1 at pH 7.5. (**b**) IC_{50} curves of Inhibitor 1 at pH 6.5. (**c**) IC_{50} curves of UNC10201652 at pH 7.5. (**d**) IC_{50} curves of UNC10201652 at pH 6.5.

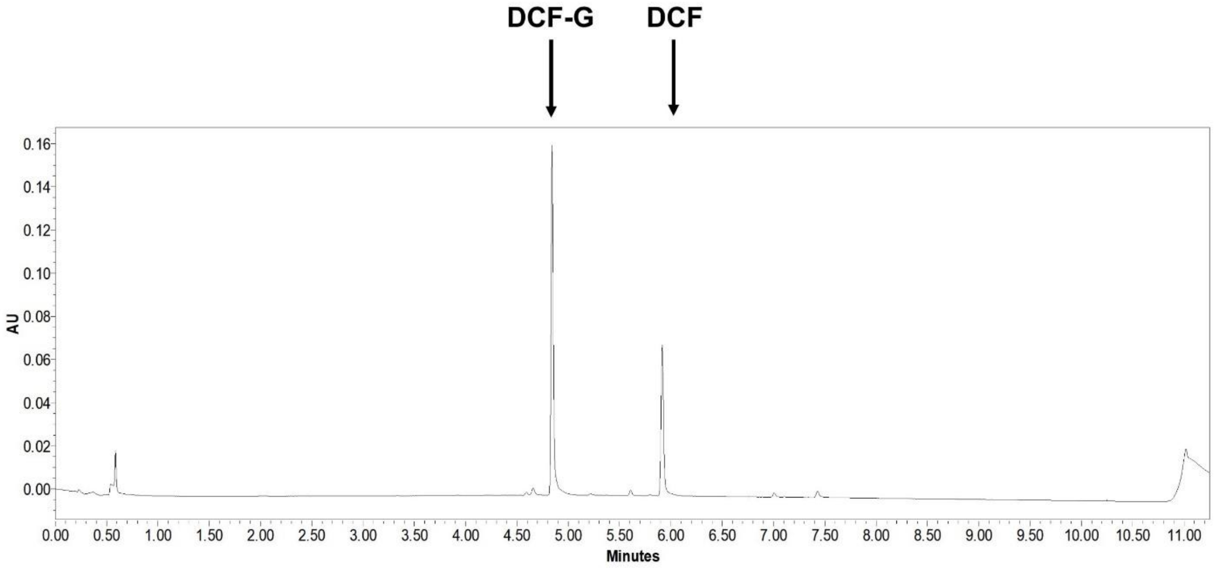

Figure S11. UPLC chromatogram following the incubation of 5 nM *Ee*GUS with 400 µM DCF-G for 3 min.

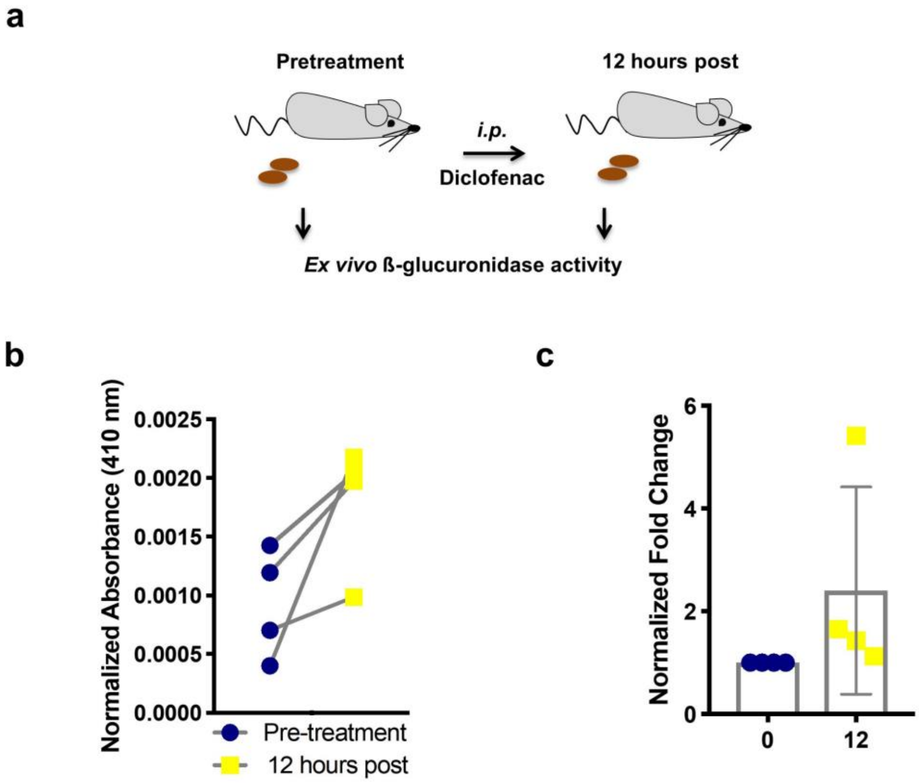

Figure S12. DCF treatment increases basal ß-glucuronidase activity *in vivo.* (**a**) Schematic of faecal collection from mice before and after 12-hour treatment with DCF. Faecal pellets were processed and analysed for ß-glucuronidase activity against *p*NPG. (**b**) Basal rates of *p*NPG cleavage increase following DCF treatment. (**c**) Data from (**b**) represented as fold change.

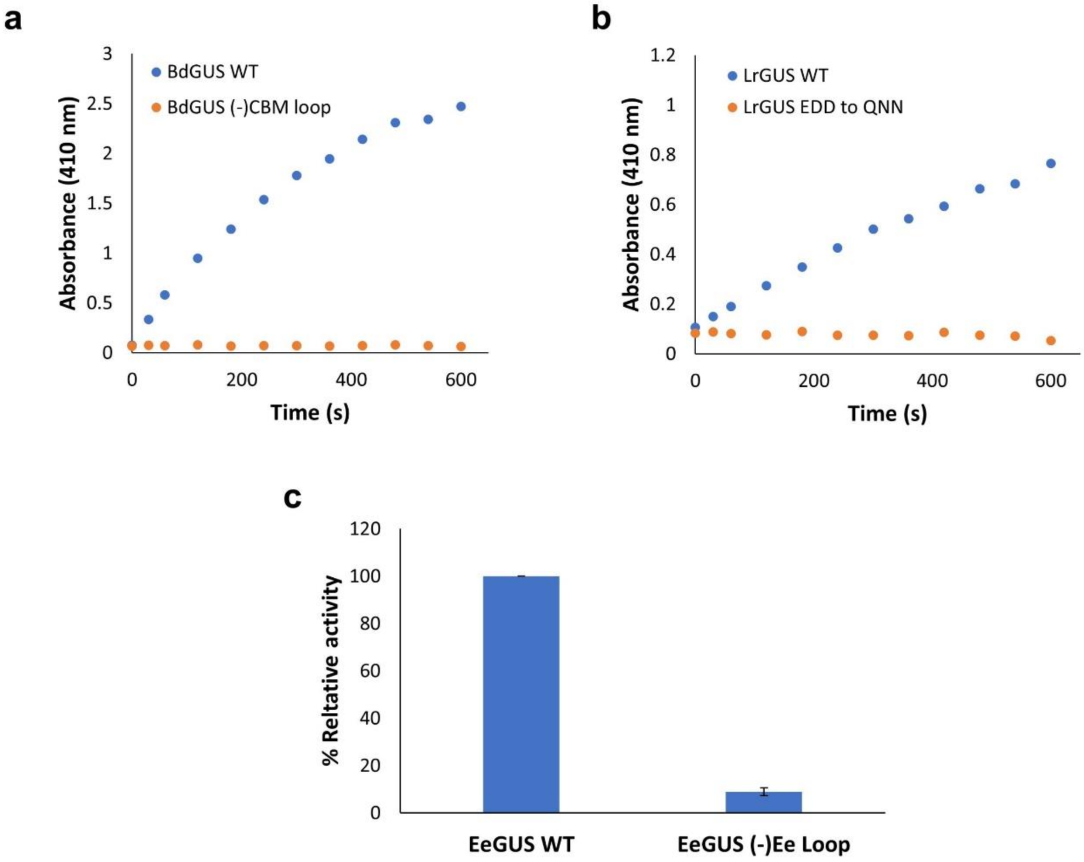

Figure S13. *p*NPG activity for WT GUS enzymes and their corresponding mutants. (**a**) Plot of absorbance versus time for WT *Bd*GUS and *Bd*GUS (-)CBM loop mutant. (**b**) Plot of absorbance versus time for WT *Lr*GUS and *Lr*GUS EDD to QNN mutant. (**c**) Relative *p*NPG activity for WT *Ee*GUS and *Ee*GUS (-)EeLoop mutant.

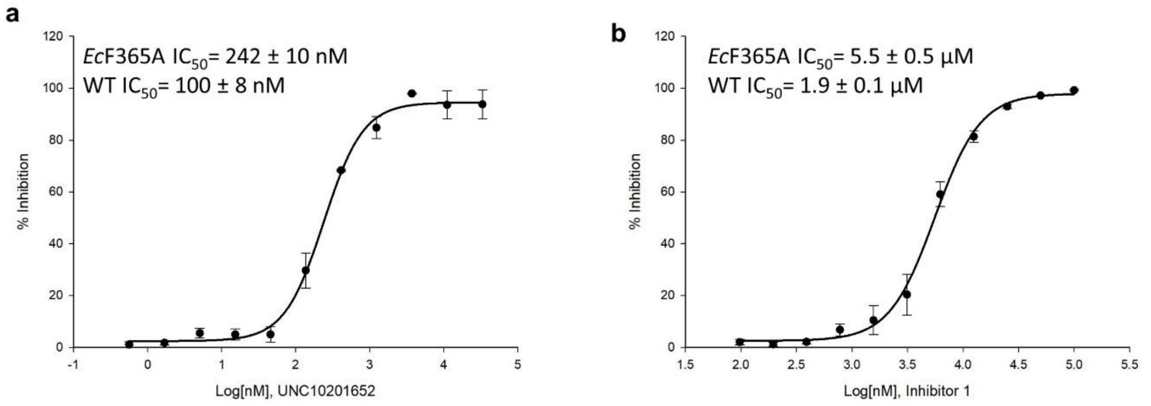

Figure S14. IC50 curves for *Ec*GUS F365A mutant with (**a**) UNC10201652 and (**b**) Inhibitor 1.

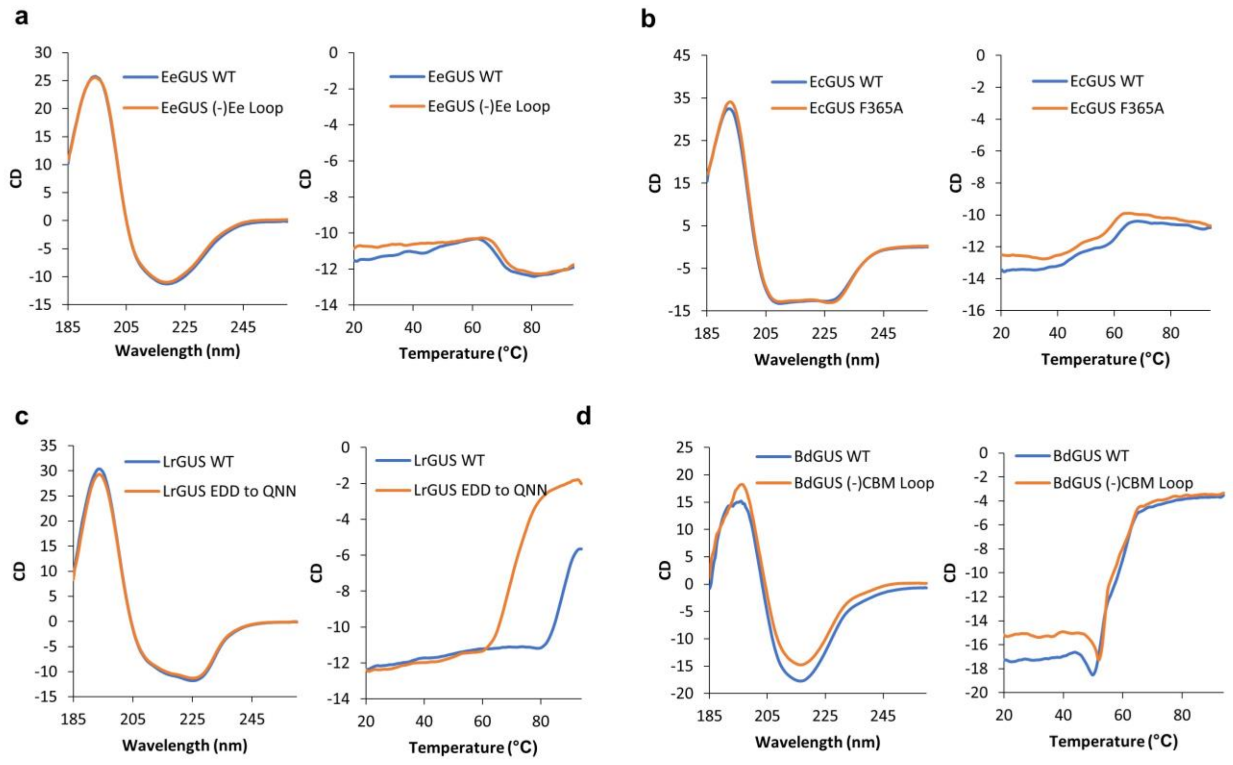

Figure S15. Circular dichroism wavelength scan and melting temperature for (**a**) *Ee*GUS WT and *Ee*GUS (-)Ee Loop mutant; (**b**) *Ec*GUS WT and *Ec*GUS F365A; (**c**) *Lr*GUS WT and *Lr*GUS EDD to QNN; and (**d**) *Bd*GUS WT and *Bd*GUS (-)CBM loop.

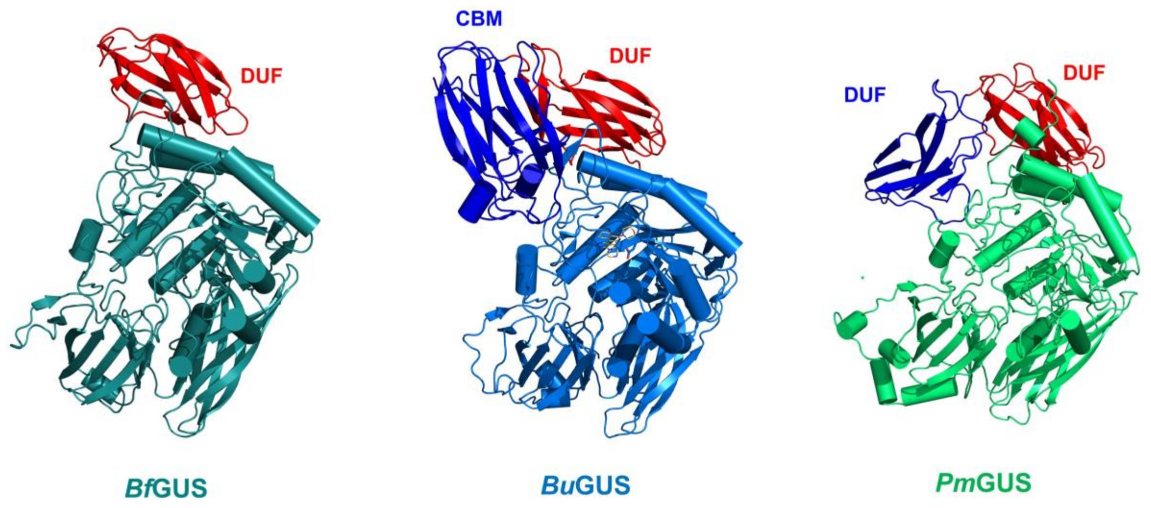

Figure S16. Monomers of *Bf*GUS (*teal*, PDB: 3CMG), *Bu*GUS (*blue,* PDB: 5UJ6), and *Pm*GUS (*light green,* PDB: 6DXU) with C-terminal domains highlighted in red and royal blue.

Table S1. Collection and refinement statistics for elucidated structures

Protein	*Lr*GUS	*Rg*GUS	*Fp*GUS	*Bd*GUS
Space group	$P6_122$	$P22_12_1$	$P6_422$	C2
Unit cell: a, b, c (Å)	154.8, 154.8, 241.9	88.3, 137.9, 153.8	145.2, 145.2, 233.9	242.6, 101.4, 168.6
Angles	90, 90, 120	90, 90, 90	90, 90, 120	90, 95.0, 90
Resolution range (Å)	29.5-2.85	29.5-2.85	28.9- 2.3	29.54-2.90
Total reflections	374,145	293,029	596,597	301,515
Unique reflections	40,232	44,603	65,087	90,006
Multiplicity	9.3 (6.1)	6.6 (6.4)	9.2 (8.4)	3.3 (3.3)
Completeness (%)	99.2 (93.9)	99.9 (100)	99.8 (100)	99.4 (99.7)
I/σ	12.6 (3.4)	15.9 (4.6)	20.2 (4.4)	9.8 (3.3)
Wilson B-factor (A^2)	36.00	38.3	33.0	33.2
R-merge	0.180 (0.510)	0.116 (0.482)	0.084 (0.516)	0.118 (0.417)
R_{work}	0.1490	0.1396	0.1717	0.1784
R_{free}	0.2125	0.1990	0.1995	0.2684
Molecules in AU	2	2	1	4
Waters in AU	187	257	356	371
Residues in AU	1182	1197	591	3440
Average B-factor	30.13	36.9	32.5	29.2
RMS (bonds)	0.008	0.015	0.009	0.009
RMS (angles)	0.921	1.249	1.240	1.060
Ramachandran favored (%)	94.63	95.29	97.61	90.91
Ramachandran outliers (%)	0.77	0.25	0.80	0.93
RCSB ID	6ECA	6EC6	6ED2	6ED1

Table S2. Primer sequences for cloning and mutagenesis studies

Primer Name	Sequence
*Lr*GUS Fwd	TACTTCCAATCCAATGCGATGGAGACATCGTTGTTATACCCAGTGAC
*Lr*GUS Rev	TTATCCACTTCCAATGCGCTACTTTGCTTTATAATCCAGCGGCAGCTTATTC
*Rg*GUS Fwd	TACTTCCAATCCAATGCGATGCTGGAATATAGCGAACTGTACCCGATCCAGAAC
*Rg*GUS Rev	TTATCCACTTCCAATGCGCTAGAACAGTTCGTTTTTCTTTTCCCAGCGATCTTTGAAAAC
*Fp*GUS Fwd	TACTTCCAATCCAATGCGATGAACCGTAGCCTGCTGTACCCTCGTGC
*Fp*GUS Rev	TTATCCACTTCCAATGCGCTATTTTTTGCGTTTTTTGAAGTCAACCGGCAGCGTGGTC
*Lr*GUS-Cterm Fwd	TACTTCCAATCCAATGCGATGGAGACATCGTTGTTATACCCAGTGAC
*Lr*GUS-Cterm Rev	TTATCCACTTCCAATGCGCTCTTTGCTTTATAATCCAGCGGCAGCTTATTC
*Fp*GUS-Cterm Fwd	TACTTCCAATCCAATGCGATGAACCGTAGCCTGCTGTACCCTCGTG
*Fp*GUS-Cterm Rev	TATCCACTTCCAATGCGCTTTTTTTGCGTTTTTTGAAGTCAACCG
*Ee*GUS (-)EeLoop Fwd	CCAGTAGGTGGTAAAGCTAACGGTGCATCTGATAAACCGCAG
*Ee*GUS (-)EeLoop Fwd	CTGCGGTTTATCAGATGCACCGTTAGCTTTACCACCTACTGG
*Bd*GUS (-) CBM Loop Fwd	TATATGGACGGCAAACCGTTCGGCGTTGGCGCGGATATTAAA
*Bd*GUS (-) CBM Loop Rev	TTTAATATCCGCGCCAACGCCGAAGCCTTTGCCGTCCATATA
*Lr*GUS EDD to QNN Fwd	ACTTTTGACGTCTCGATCGTATTATTCTGGCTCAAAGTAAACGTCCGCGGG
*Lr*GUS EDD to QNN Rev	CCCGCGGACGTTTACTTTGAGCCAGAATAATACGATCGAGACGTCAAAAGT
*Ec*GUS F365A Fwd	CTTGTTGCCCGCTTCGGCACCAATGCCTAAAGAGAGGT
*Ec*GUS F365A Rev	ACCTCTCTTTAGGCATTGGTGCCGAAGCGGGCAACAAG
